# Single-cell transcriptome atlas of lung adenocarcinoma featured with ground glass nodules

**DOI:** 10.1038/s41421-020-00200-x

**Published:** 2020-10-06

**Authors:** Tao Lu, Xiaodong Yang, Yu Shi, Mengnan Zhao, Guoshu Bi, Jiaqi Liang, Zhencong Chen, Yiwei Huang, Wei Jiang, Zongwu Lin, Junjie Xi, Shuai Wang, Yong Yang, Cheng Zhan, Qun Wang, Lijie Tan

**Affiliations:** 1 Department of Thoracic Surgery, Zhongshan Hospital, Fudan University, Shanghai, 200032 China; 2 Department of Cardio-Thoracic Surgery, First Affiliated Hospital of Soochow University, Suzhou, Jiangsu 215006 China; 3 Department of Thoracic Surgery, Suzhou Hospital affiliated to Nanjing Medical University, Suzhou, Jiangsu 215001 China

**Keywords:** Non-small-cell lung cancer, Tumour heterogeneity

## Abstract

As an early type of lung adenocarcinoma, ground glass nodule (GGN) has been detected increasingly and now accounts for most lung cancer outpatients. GGN has a satisfactory prognosis and its characteristics are quite different from solid adenocarcinoma (SADC). We compared the GGN adenocarcinoma (GGN-ADC) with SADC using the single-cell RNA sequencing (scRNA-seq) to fully understand GGNs. The tumor samples of five patients with lung GGN-ADCs and five with SADCs underwent surgery were digested to a single-cell suspension and analyzed using 10× Genomic scRNA-seq techniques. We obtained 60,459 cells and then classified them as eight cell types, including cancer cells, endothelial cells, fibroblasts, T cells, B cells, Nature killer cells, mast cells, and myeloid cells. We provided a comprehensive description of the cancer cells and stromal cells. We found that the signaling pathways related to cell proliferation were downregulated in GGN-ADC cancer cells, and stromal cells had different effects in GGN-ADC and SADC based on the analyses of scRNA-seq results. In GGN-ADC, the signaling pathways of angiogenesis were downregulated, fibroblasts expressed low levels of some collagens, and immune cells were more activated. Furthermore, we used flow cytometry to isolate the cancer cells and T cells in 12 GGN-ADC samples and in an equal number of SADC samples, including CD4^+^ T and CD8^+^ T cells, and validated the expression of key molecules by quantitative real-time polymerase chain reaction analyses. Through comprehensive analyses of cell phenotypes in GGNs, we provide deep insights into lung carcinogenesis that will be beneficial in lung cancer prevention and therapy.

## Introduction

Ground glass nodules (GGNs) are defined radiologically as focal lesions through which the lung parenchyma structures, airways, and vessels are visually preserved^1^. With the development of thoracic thin slice computed tomography (CT), lung GGNs have been detected increasingly in recent years^2^. At present, most of the lung cancer outpatients are GGN patients in Asia. According to the new IASLCA/ATS/ERS classification of lung adenocarcinoma^3^, GGNs are likely to be preinvasive lesions (atypical adenoma hyperplasias and in situ adenocarcinomas) and minimally invasive adenocarcinomas. GGNs that persist for years present slow growth^4^. A follow-up study on GGNs reported that 208/351 GGNs remained stable within the first 5 years, and even after 5 years, only 27 GGNs showed signs of growth, while no deaths were found in the cohort during the long-term follow-up^5^. Overall, GGNs have low malignant potential and present a good prognosis, which is between benign lesions and malignant tumors. However, although we observed that the GGNs were characterized by slow growth, the mechanism remains unknown. Thus, exploring the characteristics of GGNs is very helpful in understanding the mechanism of the occurrence and development of lung cancer.

Recently, single-cell RNA sequencing (scRNA-seq) techniques have been developed that enable the profiling of single cells using next-generation sequencing, which offers an unbiased single cell transcriptome profiling of a cell and its genetic heterogeneity^6^. Cancer cells are embedded in the tumor microenvironment (TME), in a complex ecosystem of stromal cells, including immune cells, fibroblasts, and endothelial cells. In the present study, using a high-throughput scRNA-seq technique, we determined the similarities and differences of cancer cells and the TME between GGNs featured with adenocarcinomas (GGN-ADCs) and solid adenocarcinomas (SADCs), and elucidated why GGN-ADCs remain stable and are associated with better survival.

## Results

### The scRNA-seq and cell types of lung GGN-ADC and SADC

Five patients with lung GGN-ADCs (Supplementary Fig. S1a–e) and an equal number of patients with SADCs underwent resection (Supplementary Fig. S1f–j). The average age of patients in the GGN-ADC and SADC groups were 63.8 and 65.4 years of age, respectively. The tumor size of GGN-ADCs was smaller than that of SADC (1.70 cm vs 2.54 cm). KI-67 expression is closely related to cell proliferation in lung cancer^7^. Immunohistochemical analyses showed that the KI-67 expression level of GGN-ADC was lower than that of SADC (8.4% vs 18.0%). Other clinical characteristics of the patients and the details of tumors are presented in Table 1.

**Table 1:** The characteristics of the GGN-ADC patients and SADC patients.

Category	Age	Sex	Tumor size	Location	Pathology	Stage	KI-67	Gene mutation
SADC 1	63	Female	2.5 cm	RUL	IAC	IB	10%	EGFR - Insertion mutation (20ins) in Exon 20
SADC 2	56	Male	2.0 cm	LLL	IAC	IB	20%	EGFR - Point mutation (L858R) in Exon 21
SADC 3	71	Male	2.2 cm	LLL	IAC	IB	10%	EGFR - Deletion mutation (19del) in Exon 19
SADC 4	67	Female	2.8 cm	RLL	IAC	IB	40%	EGFR - Deletion mutation (19del) in Exon 19
SADC 5	70	Male	3.3 cm	LUL	IAC	IB	10%	EGFR - Deletion mutation (T790M) in Exon 20, point mutation (L858R) in Exon 21
GGN 1	75	Male	1.5 cm	LUL	MIA	IA	10%	EGFR - Point mutation (L858R) in Exon 21
GGN 2	64	Female	1.7 cm	RUL	MIA	IB	5%	EGFR - Point mutation (L858R) in Exon 21
GGN 3	58	Female	2.5 cm	RML	IAC	IB	20%	EGFR - Deletion mutation (19del) in Exon 19
GGN 4	63	Male	1.6 cm	LUL	IAC	IA	5%	EGFR - Point mutation (L858R) in Exon 21
GGN 5	59	Female	1.2 cm	RUL	MIA	IA	2%	HER-2 - Heterozygous duplication of codons 771-774 in Exon 20

RUL right upper lobe, LLL left lower lobe, RLL right lower lobe, LUL left upper lobe, RML right middle lobe, IAC invasive adenocarcinoma cancer, MIA minimally invasive adenocarcinoma

**Fig. 1:**
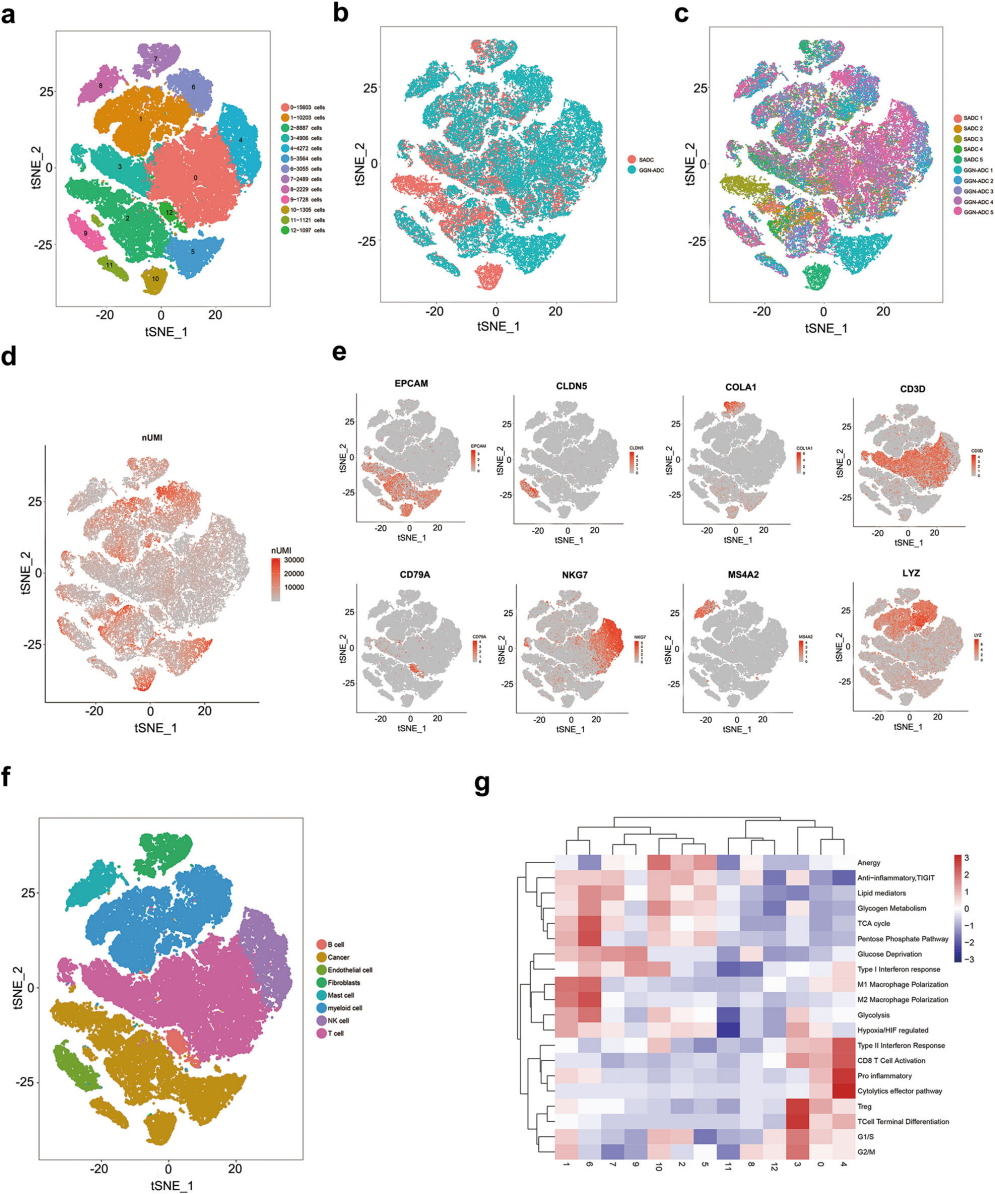
Overview of the 60,459 single cells from five GGN-ADC samples and five SADC samples. **a** tSNE plotting of the 60,459 cells showing 13 cell clusters; **b** The sample origin of the cells; **c** The patient origin of the cells; **d** The nUMI detected in each cell; **e** Expression of marker genes for the cell types; **f** The cell types identified by marker genes; **g** Transcriptomic signatures mean heatmap of each cluster.

The tumor samples were digested to a single-cell suspension and analyzed using scRNA-seq techniques. After quality filtering, 60,459 cells were obtained from ten tumor samples. Quality control metrics were showed in Supplementary Fig. S2. Also, the distribution of the percentage of mitochondrial counts was shown in Supplementary Fig. S3. Of these cells, 34,285 cells (56.02%) were derived from GGN-ADC samples and 26,174 cells (43.98%) were derived from SADC samples. All cells were classified as 13 clusters (Fig. 1a). However, these clusters failed to show separation between GGN-ADC and SADC tissues on t-distributed stochastic neighbor embedding (tSNE) plotting, indicating that both GGN-ADC and SADC tissues contributed to each of the cell types (Fig. 1b and Supplementary Fig. S4a). With the exceptions of clusters 5, 10, and 11, each cluster contained cells derived from all ten samples (Fig. 1c and Supplementary Fig. S4b). Cluster 5 mainly originated from the GGN-ADC-1 patient, cluster 10 completely originated from SADC-5 patient, and cluster 11 originated from all patients except the SADC-4 patient. The number of unique molecular identifier (nUMI) of each cell is shown in Fig. 1d. Based on the marker genes, we identified the cells as eight cell types (Fig. 1e–f), namely cancer cells (EPCAM; clusters 2, 5, 10, and 11), fibroblasts (COL1A1; cluster 7), endothelial cells (CLDN5; cluster 9), T cells (CD3D; clusters 0 and 3), B cells (CD79A; cluster 12), NK cells (NKG7; cluster 4), mast cells (MS4A2; cluster 8), and myeloid cells (LYZ; clusters 1 and 6). The transcriptomic signatures cluster mean heat map showed the enriched functions of each cluster (Fig. 1g). Overall, this analysis showed the presence of a complex cellular ecosystem. We identified 50 different stromal cell subclusters (including 7 clusters from endothelial cells, 5 clusters from fibroblasts, 17 clusters from T cells, 3 cluster from B cells, 4 cluster from NK cells, 11 clusters from macrophages, and 3 clusters from mast cells) and 13 cancer cell subclusters, of which, we focused on some important cell subclusters to explore their functions.

### Signaling pathways related to cell proliferation were downregulated in GGN-ADC cancer cells

**Fig. 2:**
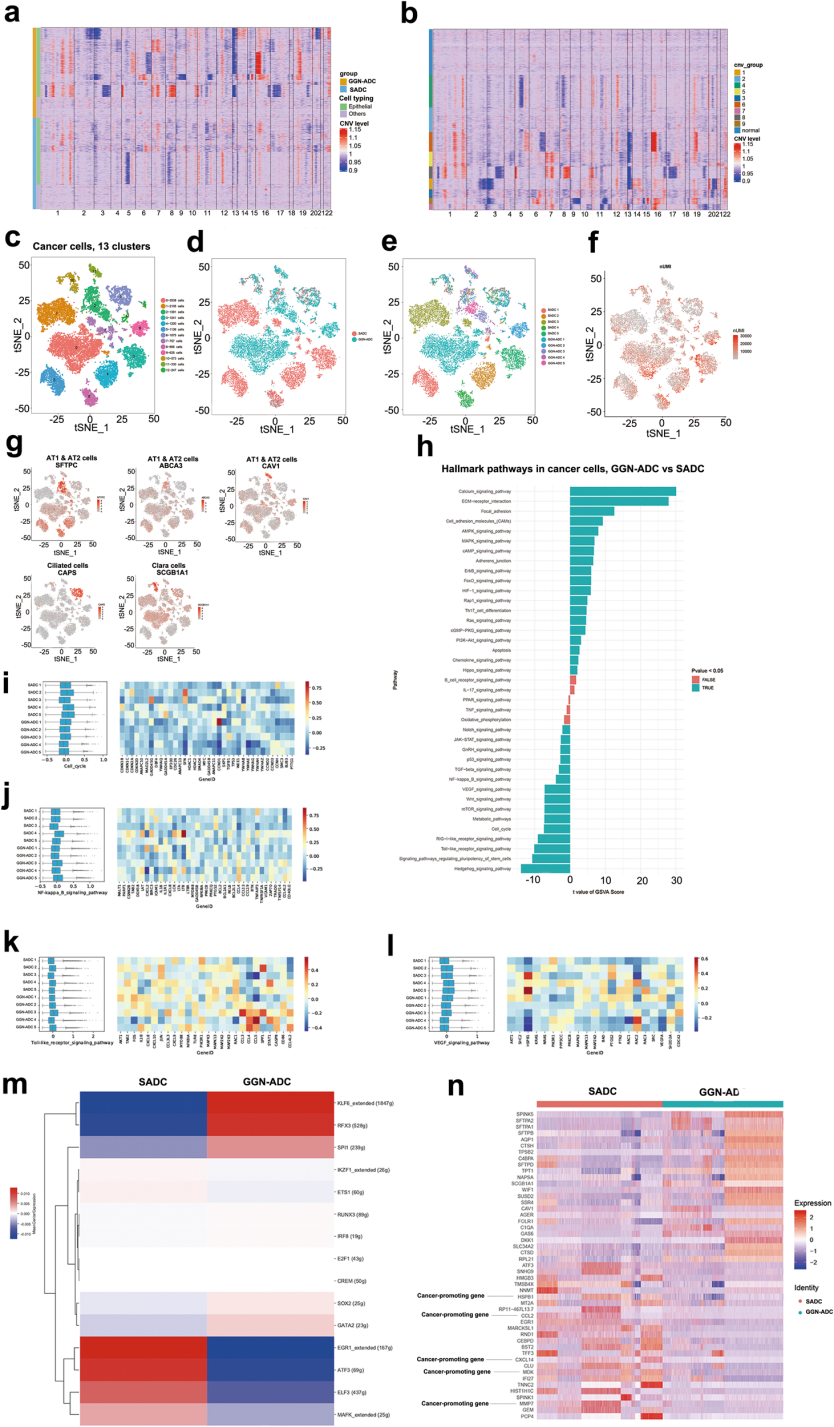
The profile of 14,877 cancer cells. **a** The heatmap of the relative expression density of genes on each chromosome between SADC and GGN-ADC groups; **b** The heatmap of the relative expression density of genes on each chromosome by comparing the relative expression density of the genes in the tumor cell genome with a series of normal cell reference genomes. **c** tSNE plotting of the 14,877 cells revealing 13 clusters; **d** The sample origin of the cancer cells; **e** The patient origin of the cancer cells; **f** The nUMI detected in each cell; **g** Expression of marker genes for the cell types; **h** GSVA analysis revealing the hallmark pathways in cancer cells between GGN-ADC derived and SADC-derived cancer cells; **i–l** The significantly changed signaling pathways and genes expression in each sample; **m** SCENIC analysis of the cancer cells (GGN-ADC vs SADC); **n** The heatmap of differential gene expression in GGN-ADC and SADC.

We considered the cells marked by EPCAM were cancer cells referred to a scRNA-seq study of lung cancer^8^. Furthermore, based on CNV analysis, we showed that the extensive deletion along chromosome 13 was shown in SADC samples (Fig. 2a). Compared to normal cells at the bottom of each of the SADC and GGN-ADC groups in Fig. 2a (including endothelial cells, fibroblasts, T cells, B cells, NK cells, myeloid cells, and mast cells), according to cell chromosome fragment variation, the cells marked by EPCAM were clustered into nine groups. We showed the relative expression density of genes on each chromosome, namely chromosome deletion or amplification. The amplification along chromosome 2 was evident even in group 2 (Fig. 2b). Therefore, most of the EPCAM-positive cells were likely cancer cells due to the presence of copy number changes.

The 14,877 cancer cells detected in 10 samples were clustered into 13 distinct subsets (Fig. 2c). We showed the cluster-to-cluster distance of cancer cells (Supplementary Fig. S5). Among them, the number of GGN-ADC-derived cancer cells was 7295 (49.04%), while the number of SADC-derived cells was 7582 (50.96%). Cancer cells accounted for 21.28% of all GGN-ADC-derived cells, which was less than the 28.97% of SADC-derived cells. The clusters showed a group specificity (Fig. 2d). Clusters 0, 2, 9, 11, and 12 were mostly enriched in GGN-ADC, while clusters 1, 3, 4, 5, and 8 were overall enriched in SADC (Supplementary Fig. S6). The group-by-group tSNE showed that the clusters from SADC were highly patient-specific. Five SADC clusters (clusters 1, 3, 4, 5, and 8) were derived from five different SADC patients, indicating the greater heterogeneity of malignancy in SADC cells compared to GGN-ADC cells (Fig. 2e). The nUMI of each cancer cell was shown in Fig. 2f. These 13 clusters, according to their novel markers, showed mixed AT1 and AT2 cells (SFTPC, ABCA3, AGER: clusters 0–5, 7–9, and 11–12), ciliated cells (CAPS: cluster 6), and Clara cells (SCGB1A1: cluster 10) (Fig. 2g). The AT1 and AT2 cells could not be distinguished clearly based on novel markers. Both GGN-ADC and SADC samples contributed to the components of all three cell subtypes.

Gene Set Variation Analysis (GSVA) analyses showed that some signaling pathways were differentially regulated when comparing GGN-ADC-derived with SADC-derived cancer cells (Fig. 2h). The downregulated genes in GGN-ADC-derived cancer cells were mostly significantly enriched in the Hedgehog signaling pathway components. Moreover, the downregulated pathways included the cell cycle (Fig. 2i), NF-κB signaling pathway (Fig. 2j), Toll-like receptor (TLR) signaling pathway (Fig. 2k), and vascular endothelial growth factor (VEGF) signaling pathway (Fig. 2l) (all *P* < 0.001). We analyzed the signaling pathway changes and the gene expression changes in each sample. TLR4/NF-κB signaling pathway activation could promote migration and invasiveness of multiple cancers^9,10^. The genes, JUN, involved in the TLR signaling pathway, and ICAM1, involved the NF-κB signaling pathway, were significantly downregulated in GGN-ADC samples. Cell cycle arrest of GGN-ADC cells showed cell cycle arrest compared with SADC-derived cells, in which SFN and GADD45G were poorly expressed in GGN-ADC cells. VEGF signaling pathway downregulation indicated that the angiogenesis capacity of GGN-ADC was relatively weaker than that of SADC, and the expression of HSPB1, BAD, and VEGFA in GGN-ADC cells was downregulated.

Single-Cell Regulatory Network Inference and Clustering (SCENIC) analyses showed that KLF6 was most significantly upregulated, while EGR1 was most significantly downregulated in GGN-ADC-derived cancer cells when compared with SADC cancer cells based on the area under the curve (AUC) scores (Fig. 2m). KLF6 was a tumor suppressor gene that was functionally inactivated in NSCLC by loss of heterozygosity, somatic mutation, decreased expression, and increased alternative splicing into an oncogenic splice variant^11^. EGR1 had been shown to regulate genes influencing proliferation, apoptosis, immune cell activation, and matrix degradation, and so on. EGR1 levels increased in response to suppressed androgen receptor signaling or loss of the tumor suppressor, PTEN^12^.

Heatmap of differential gene expression in GGN-ADC and SADC showed that some cancer-promoting genes, such as HSPB1, CCL2, CXCL14, MDK, and MMP7, were highly expressed in SADC, indicating that the cancer cells derived from SADC had a higher malignant degree than those derived from GGN-ADC (Fig. 2n).

### The signaling pathways of angiogenesis were downregulated in GGN-ADC endothelial cells

**Fig. 3:**
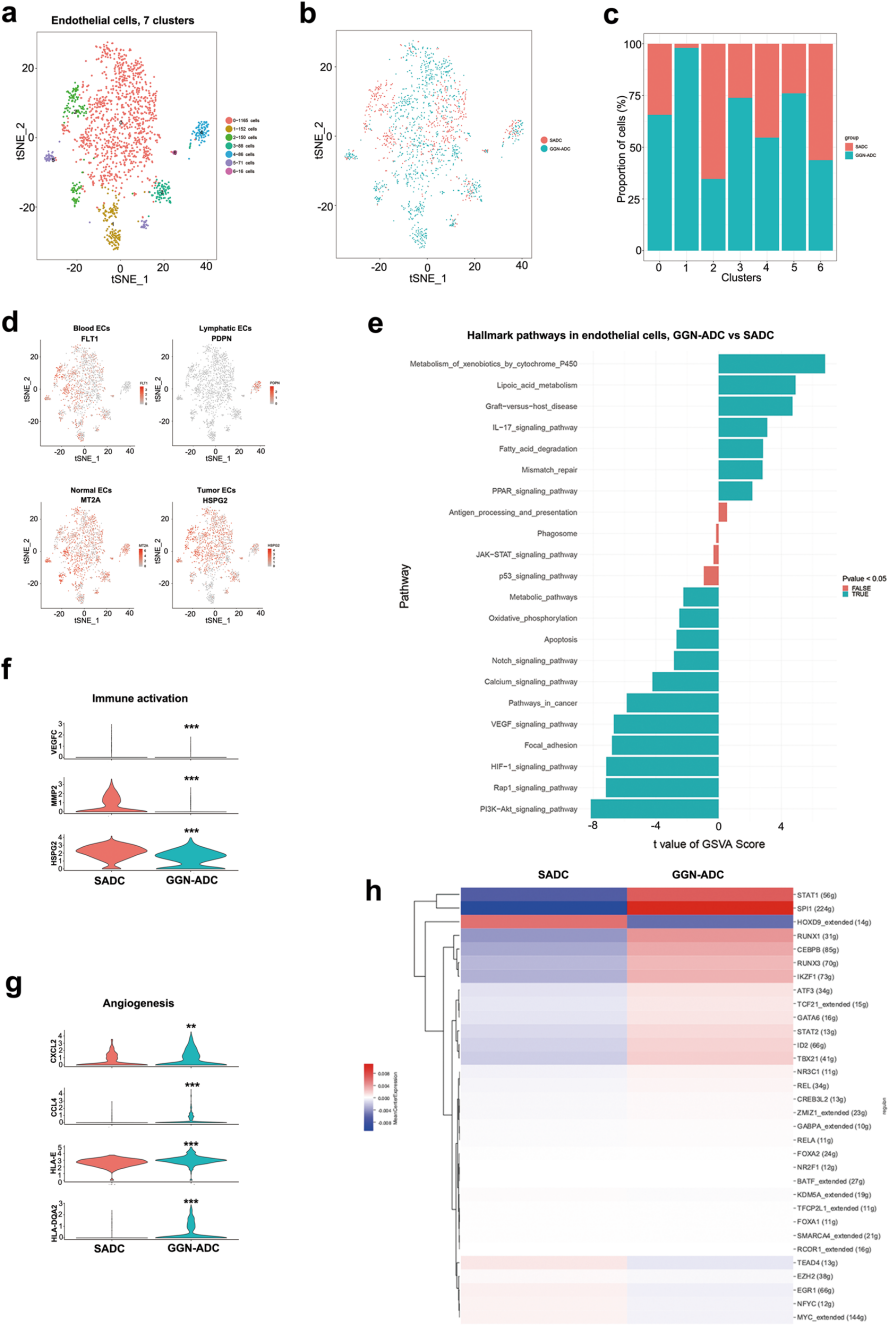
Endothelial cells clusters. **a** tSNE plotting of 1728 endothelial cells revealing seven clusters; **b**, **c** The sample origin of endothelial cells; **d** Expression of marker genes identified the endothelial cells as blood endothelial cells, lymphatic endothelial cells, normal endothelial cells and tumor endothelial cells; **e** GSVA analysis of the hallmark pathways in blood endothelial cells between GGN-ADC and SADC; **f**, **g** The key genes expression of angiogenesis and immune activation in blood endothelial cells (GGN-ADC vs SADC); **h** SCENIC analysis of blood endothelial cells between GGN-ADC and SADC.

Endothelial cells only accounted for 2.86% (1728) of all detected cells. There were no significant differences in the numbers of endothelial cells between GGN-ADC and SADC samples (3.42% vs 2.24%; *P* = 0.269). Reclustering these endothelial cells revealed seven clusters (Fig. 3a). All these cells were mixed together, so it was not possible to distinguish between GGN-ADC and SADC cells (Fig. 3b), apart from cluster 1 that was mainly derived from GGN-ADC tissues (Fig. 3c). The cells derived from cluster 0 contributed to more than two-thirds of all endothelial cells. The patient origin of the endothelial cells was shown in Supplementary Fig. S7. Five clusters contained >100 cells, especially cluster 6, which only included 16 cells. According to the marker genes, these clusters were designated as blood endothelial cells (FLT1; clusters 0–3, 5, 6), lymphatic endothelial cells (PDPN: cluster 4), tumor endothelial cells (HSPG2: clusters 0 and 2–6), and normal endothelial cells (MT2A; clusters 0–6) (Fig. 3d).

Angiogenesis is a hallmark of cancer and plays a critical role in lung cancer progression, which involves interactions between cancer cells, endothelial cells, and the surrounding microenvironment. In this study, we focused on differences of the blood endothelial cells between the two groups. GSVA analyses of hallmark pathways revealed that the PI3K/AKT signaling pathway was most significantly downregulated in GGN-ADC cells (Fig. 3e). The PI3K/AKT pathway is activated in the majority of human cancers, and activation of PI3K/AKT signaling pathway could stimulate angiogenesis by mediating VEGFR2/VEGF-A overexpression^13^. Some other signaling pathways involving angiogenesis, such as the HIF-1 signaling and VEGF signaling pathways were also downregulated, indicating that the angiogenic ability of blood endothelial cells in GGN-ADC cells were weaker than those in SADC cells. In addition, the downregulation of metabolic pathways and oxidative phosphorylation suggested that the metabolism of blood endothelial cells derived from GGN-ADC tissues was relatively inactive in comparison with those of SADC.

We showed that the expression level of VEGFC in GGN-ADC cells was significantly less than that in SADC cells (fold change = 0.64, *P* < 0.001), in spite of their low expression levels. Other genes involved in angiogenesis, such as MMP2 and HSPG2, were downregulated more in GGN-ADC cells than SADC cells (*P* < 0.001; Fig. 3f). In addition, the genes related with antigen presentation (HLA-E and HLA-DQA2) and chemotaxis (CCL4 and CXCL2), showed upregulation in GGN-ADC cells (all *P* < 0.05; Fig. 3g). Taken together, these results indicated that blood endothelial cells induced the immune activation in GGN-ADC.

Finally, using SCENIC analyses, we determined which transcription factors were involved in differences in expression between GGN-ADC-derived and SADC-derived blood endothelial cells (Fig. 3h). According to the AUC scores, HOXD9 was the most significantly downregulated transcriptional factor, which regulated the expressions of 14 genes, while SPI1 and RUNX1 were the most significantly upregulated transcriptional factors, which regulated 244 and 31 genes, respectively. HOXD9 was considered to promote the growth, invasion, metastasis of cancer cells^14^. The transcription factor RUNX1 cooperated with lineage-specifying TFs (e.g., PU.1/SPI1) to activate myeloid differentiation genes, such as macrophage^15^. RUNX1 acted as an inducer of DNA demethylation at the SPI1 regulatory regions^16^. Notably, Lis et al. demonstrated that the endothelium could be converted to immunocompetent haematopoietic stem cells through transient expression of the transcription-factor-encoding genes Fosb, Gfi1, RUNX1, and SPI1^17^.

### Fibroblasts derived from GGN-ADC expressed low levels of some collagens

**Fig. 4:**
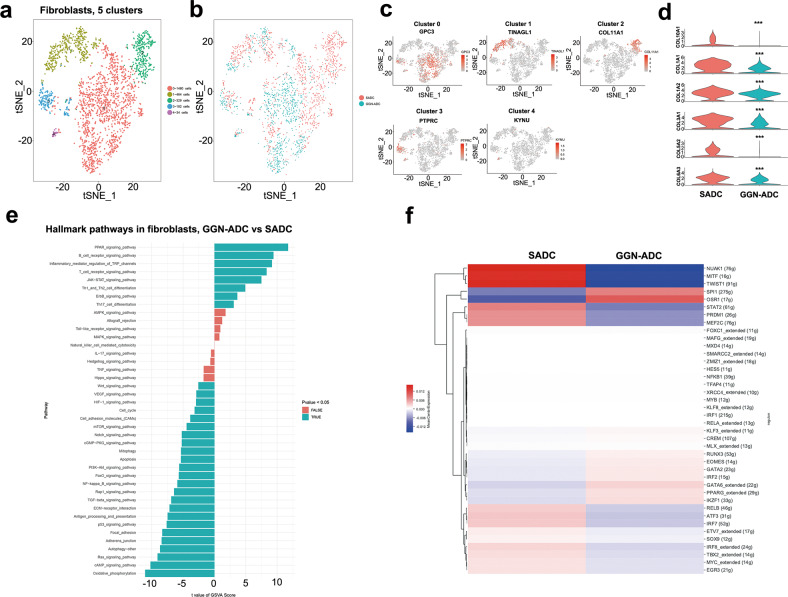
The profile of 2489 fibroblasts derived from GGN-ADC and SADC. **a** tSNE plotting of fibroblasts revealing 5 clusters; **b** The sample origin of the cells; **c** Expression of marker genes of the clusters; **d** The differential expression of key genes in fibroblasts between GGN-ADC and SADC; **e** GSVA analysis revealing the hallmark pathways in fibroblasts (GGN-ADC vs SADC); **f** SCENIC analysis of fibroblasts between GGN-ADC and SADC.

In the current study, 2489 fibroblasts were detected. Among them, 1073 (43.11%) of the cells derived from GGN-ADC tissues and 1416 (56.89%) were cells derived from SADC tissues. These cells are reclustered as five clusters (cluster 0, GPC3; cluster 1, TINAGL1; cluster 2, COL11A1; cluster 3, PTPRC; and cluster 4, KYNU) (Fig. 4a, c). All other clusters were derived from both GGN-ADC and SADC tissues (Fig. 4b and Supplementary Fig. S8a). Cluster 2 was strongly enriched in one SADC patient (Fig. S8b, c). Compared with SADC cells, GGN-ADC derived fibroblasts expressed low levels of some collagens (COL1A1, COL1A2, COL3A1, COL5A2, COL6A3, and COL10A1) (all *P* < 0.001; Fig. 4d).

GSVA analyses showed that oxidative phosphorylation was the most significantly downregulated pathway in GGN-ADC cells, followed by the cAMP signaling pathway, suggesting that the cell metabolism of GGN-ADC derived fibroblasts was inhibited, when compared with that of SADC-derived cells (Fig. 4e). The reduction of cell metabolic activity may be related with the decreased synthesis and secretion of collagens. Moreover, the Ras-PI3K-mTOR signaling pathway, which plays an important role in regulating cell growth, proliferation, and glucose metabolism, was also significantly downregulated^18^.

Analyses of gene expression showed that MMP11 was the most downregulated gene, and SCGB3A1 was the most significantly upregulated gene in GGN-ADC cells (Supplementary Fig. S8d). Although MMP-11 does not degrade the main components of extracellular matrix^19^, it has been shown to be involved in interstitial remodeling and tumorigenesis^20,21^. MMP-11 is abundantly produced in lung cancer interstitial fibroblasts. SCGB3A1 is a secretory protein of small molecular weight, which had been reported as a potential tumor suppressor^22^. Higher expression of SCGB3A1 in GGN-ADC derived fibroblasts may play an important role in cancer cells, which needed to be validated by experiments.

The SCENIC analyses showed that PPARG was most significantly upregulated and SOX9 and EGR3 were most significantly downregulated (Fig. 4f). The protein encoded by PPARG was a regulator of adipocyte differentiation. Additionally, PPARG had been implicated in the pathology of numerous diseases including obesity, diabetes, atherosclerosis and cancer. PPARG was involved in controlling angiogenesis, tumor progression and metastasis. Upregulation of PPARG inhibited the movement and angiogenesis of cancer cells^23^. SOX9 could significantly upregulate the proliferation rate and inhibit apoptosis rate and inflammatory factor expression in MRC-5 cells (lung fibroblast cell line)^24^. EGR3 played a role in cell growth and migration. It had been reported that EGR3 depletion resulted in a significant decrease of migratory and invasive abilities of CL1-5 cells^25^.

### The immunosuppressive pathways were activated in T cells derived from GGN-ADC

**Fig. 5:**
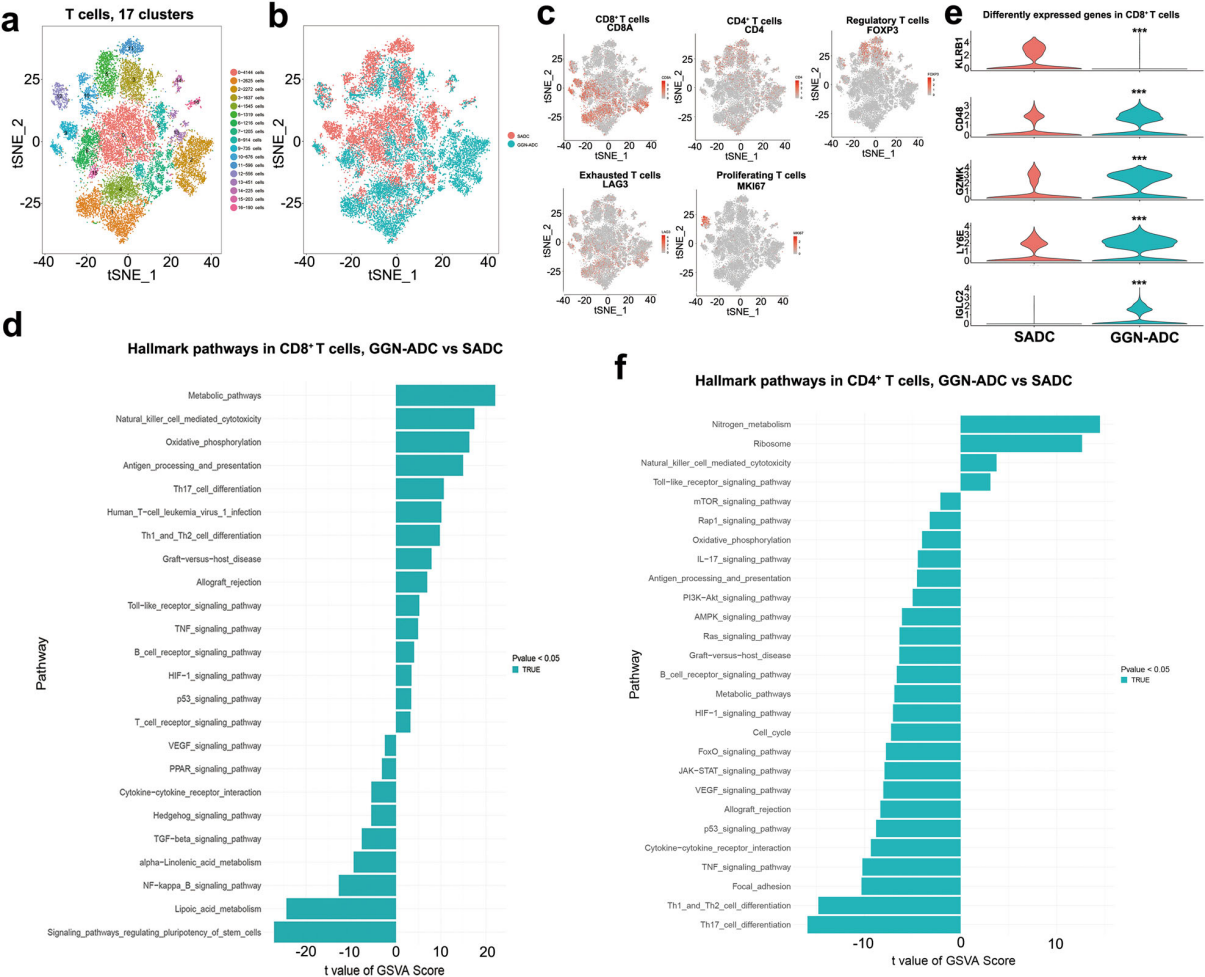
The role of the T cells in the immune functions of GGN-ADC and SADC. **a** tSNE plotting of 20,509 T cells revealing 17 clusters; **b** The sample origin of T cells; **c** Reclustering the T cells revealing 5 cell subtypes, including CD8+ T cells, CD4+ T cells, regulatory T cells, exhausted T cells and proliferating T cells; **d** GSVA analysis of the hallmark pathways in CD8+ T cells (GGN-ADC vs SADC); **e** The differently expressed genes in CD8+ T cells between GGN-ADC and SADC; **f** GSVA analysis of the hallmark pathways in CD4+ T cells (GGN-ADC vs SADC).

In our study, 20,509 T cells were detected, accounting for 33.92% of all cells. T cells are the most prevalent cell type. Among them, 10,547 cells were derived from the GGN-ADC sample and 9962 cells were derived from the ADC sample. The percentage of T cells in GGN-ADC cells (31.26%) was lower than that in SADC cells (38.60%), but not statistically significant (*P* = 0.582). These T cells were regrouped into 17 clusters (Fig. 5a). We showed the cluster-to-cluster distance of between SADC-related immune cells and GGN-ADC-related immune cells (Supplementary Fig. S9). The tSNE of group by group resolution showed that the clusters could not be distinguished between the GGN-ADC and SADC groups, except for clusters 2, 8, and 11 (Fig. 5b and Supplementary Fig. S10). Cluster 2 was mainly derived from GGN-ADC tissues, while cluster 8 and 11 were mainly derived from SADC tissues. These clusters were defined as CD8+ T cells (CD8A: clusters 2, 4, 6, and 9), CD4+ T cells (CD4; clusters 0, 2, 3, 5, 8, 10, 11, 14, and 16), regulation T cells (FOXP3: clusters 3, 5, and 11), proliferating T cells (MKI67: cluster 12), and exhausted T cells (LAG3: clusters 0–13 and 15) (Fig. 5c).

CD8 is expressed in 30–35% of T cells. Upon activation, CD8+ T cells differentiate into cytotoxic T cells (CTLs), which is the major functional subpopulation. CTL can specifically recognize endogenous antigenic peptides presented by the major histocompatibility complex I, thereby killing tumor cells^26^. We conducted the GSVA analyses to compare the differences of pathways between the GGN-ADC-derived and SADC-derived CD8+ T cells (Fig. 5d). The metabolic pathway and oxidative phosphorylation were significantly activated, indicating functional enhancement of CD8+ T cells derived from GGN-ADC. Also, the oxidative phosphorylation pathway was involved in allograft rejection, suggesting that targeting this pathway may enhance immunotherapy^8^. In addition, the pathway of antigen processing and presentation was upregulated in GGN-ADC derived CD8+ T cells. All these activated signaling pathways suggested that CD8+ T cells had a stronger immune suppression on GGN-ADC than SADC cancer cells.

Differential gene expression analyses of CD8+ T cells showed one downregulated gene (KLRB1) and four upregulated genes (CD48, GZMK, LY6E, and IGLC2) in GGN-ADC cells (Fig. 5e). KLRB1 plays an inhibitory role on natural killer (NK) cell cytotoxicity. The lower expression level of KLRB1 in GGN-ADC indicated the activation of NK cell-mediated cytotoxicity. CD48 and GZMK expression levels in GGN-ADC cells were higher than those in SADC, which probably involved regulating CD8+ T cell activation^27^. In parallel, GGN-ADC cells expressed higher levels of immune checkpoint molecules, including LY6E and IGLC2. All of these genes were associated with CD8+ T cell activity in GGN-ADCs, suggesting higher cytotoxic activities.

We found that the cluster 4 of CD8^+^ T cells was predominantly enriched in GGN-ADCs. We have further explored the biology of this cluster. GO analysis showed that the most enriched terms of biological process, cellular component and molecular function was nuclear-transcribed mRNA catabolic process, nonsense-mediated decay, extracellular exosome, and TAP2 binding, respectively (Supplementary Fig. S11a). KEGG analysis revealed that antigen processing and presentation was the most significantly enriched signaling pathway (Supplementary Fig. S11b).

It is well-recognized that CD4+ T cells may play an important role in immunosurveillance and immunotherapy against cancer. In our study, CD4+ T cells accounted for 41.68% in GGNs and 45.93% in SADC. GSVA analyses revealed that the Th17 cell differentiation pathway is significantly inhibited in GGNs compared with that in SADC (Fig. 5f). Previous studies showed that Th17 cells are involved in antitumor responses^28^. However, studies have also shown that Th17 cells can promote angiogenesis by secreting IL-17, and participating in tumor formation^29^. IL-17 has been shown to induce pro-angiogenic factor expression in mesenchymal, epithelial, and tumor cells, such as VEGF, angiotensin, IL-8, and prostaglandin E2^29^. Moreover, the IL-17 signaling pathway is also downregulated in GGN-ADCs. Hence, the exact cellular mechanism by which Th17 is pro-tumor or antitumor in lung adenocarcinoma is still unclear, and needs to be further studied. The Th1 and Th2 cell differentiation pathway is also downregulated in GGNs. Besides, the VEGF and HIF signaling pathways, related to angiogenesis, are also inhibited in GGN-ADCs. Notably, NK cell-mediated cytotoxicity is significantly activated, which indicates an enhancement of immune responses by NK cells.

### B cells present antigens and trigger an immune response in GGN-ADCs

**Fig. 6:**
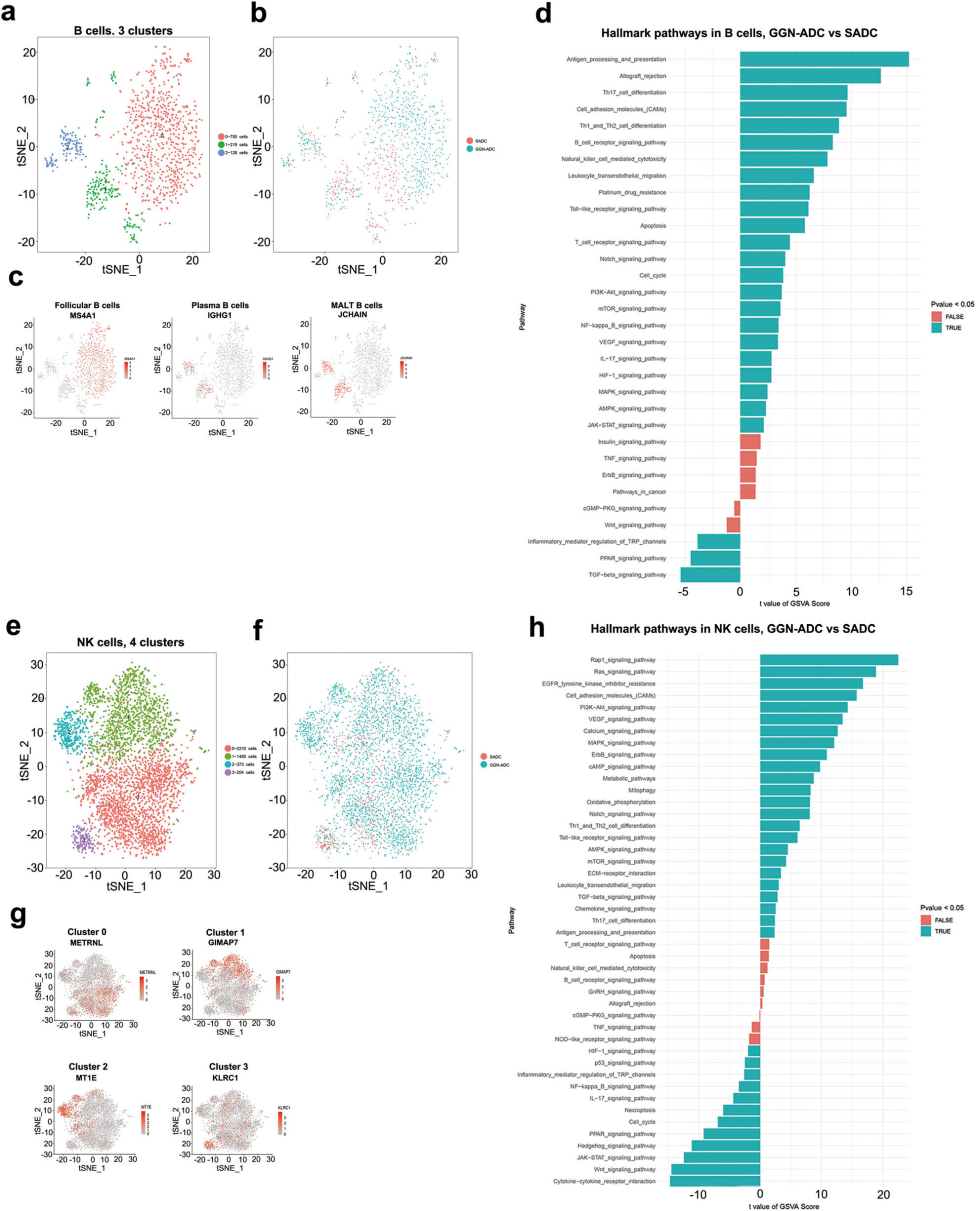
The immunoregulation effect of B cells and NK cells in GGN-ADC and SADC. **a** tSNE plotting of 1097 B cells revealing three clusters; **b** The sample origin of B cells; (**c**) Expression of marker genes of B cells; **d** GSVA analysis of hallmark pathways in B cells (GGN-ADC vs SADC); **e** tSNE plotting of 4272 NK cells revealing four clusters; **f** The sample origin of NK cells; **g** The marker gene expression of each cluster; **h** GSVA analysis of hallmark pathways in NK cells between GGN-ADC and SADC.

Only 1097 B cells were detected, accounting for 1.8% of the total cells. GGN-ADC samples processed 718 B cells (2.1%), while SADC samples processed 379 cells (1.5%). These B cells were classified into three clusters (Fig. 6a). B cells were relatively more enriched in GGN-ADCs (Fig. 6b and Supplementary Fig. S12a). The patient origin of the B cells clusters was shown in Supplementary Figs. S12b, c. Cluster 0 corresponded to follicular B cells (MS4A1), which comprised the majority of the B cells. Both cluster 1 and cluster 2 expressed high levels of IGHG1 and JCHAIN, which corresponded to plasma B cells and MALT B cells, respectively (Fig. 6c).

GSVA pathway analyses showed that the immune response was stronger in B cells derived from GGN-ADC than SADC (Fig. 6d). In addition to producing specific antibodies, B cells are also antigen presenting cells. The effect of antigen processing and presentation by B cells is greatly enhanced in GGN-ADCs. The pathway of NK cell-mediated cytotoxicity, which is involved in allograft rejection, is significantly upregulated^30^. Based on these observations, the B cells had stronger immunosuppression effects on the GGN-ADC lesions than SADC.

### The cytotoxicity of NK cells is more robust in GGN-ADCs

The 4272 NK cells were detected from ten samples, accounting for 7.1% of all cells. Reclustering these cells revealed cluster 0 (METRNL), cluster 1 (GIMAP1), cluster 2 (MT1E), and cluster 3 (KLRC1) (Fig. 6e, g). Among them, the quantity of GGN-ADC derived from NK cells was 3689 (86.4%) and SADC-derived cells was 583 (13.6%). The NK cells were the most GGN-ADC enriched cell type (Fig. 6f and Supplementary Fig. S12d). The cells of cluster 2 mainly derived from one GGN-ADC patient (Supplementary Figs. S12e, f).

GSVA analyses showed that the Rap1 signaling pathway was mostly significantly enriched in GGN-ADCs compared with SADC (Fig. 6h). Rap1b, an isoform of rap1, facilitates NK cell functions and is responsible for the generation of cytokines and chemokines^31^. PI3K/AKT also plays a pivotal role in both priming and signaling downstream of cytokine activation^32^. Metabolic and oxidative phosphorylation pathways were regulated, suggesting NK cell activation in GGN-ADCs. In addition, chemokine signaling pathway and antigen processing and presentation were activated, indicating that the immune response induced by NK cells was more robust in GGN-ADCs.

### Macrophages tend to become M1 polarized in GGN-ADCs

**Fig. 7:**
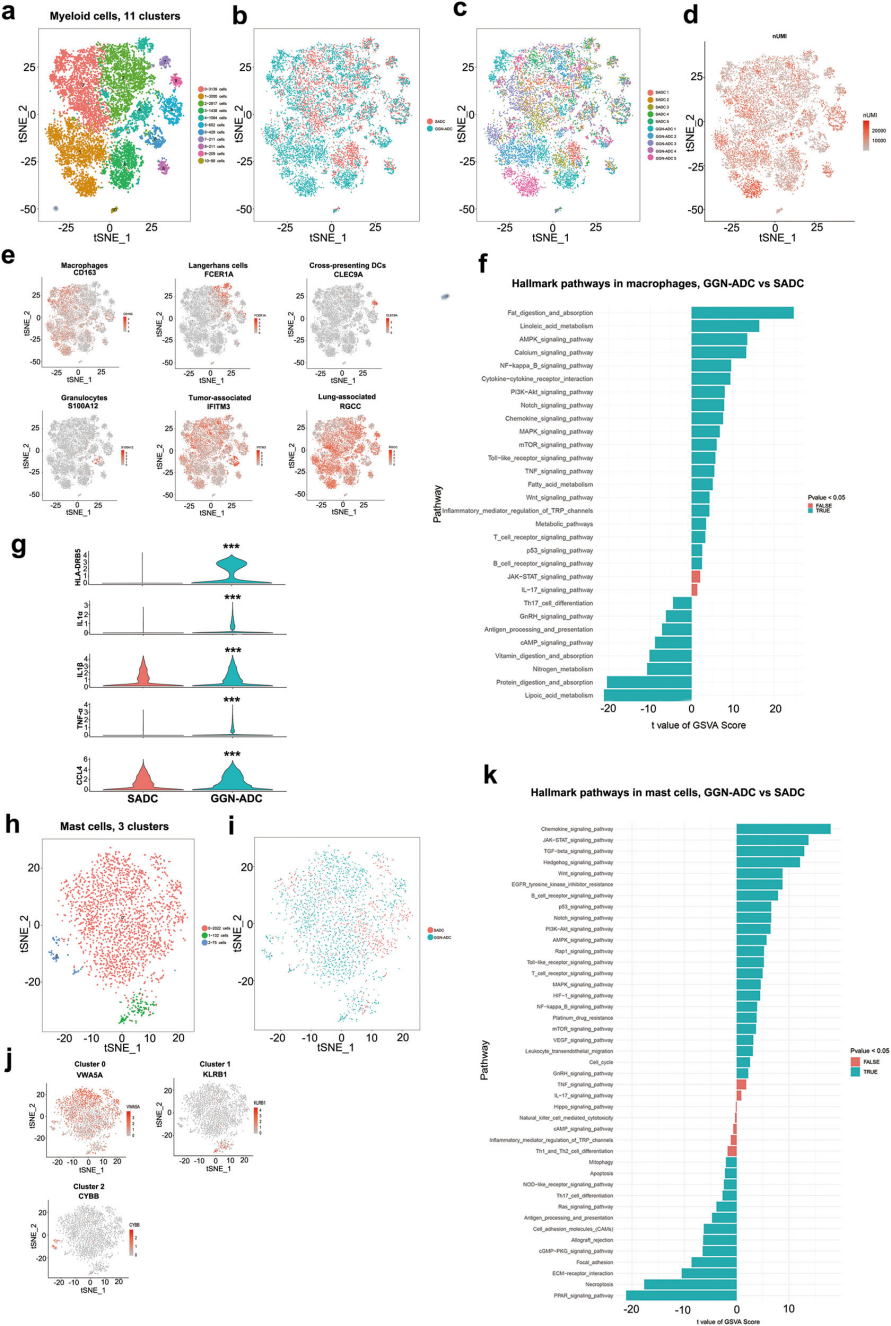
The role of myeloid cells and mast cells in immune regulation of GGN-ADC and SADC. **a** tSNE plotting of 13,258 myeloid cells revealing 11 clusters; **b** The sample origin of myeloid cells; **c** The patient origin of myeloid cells; **d** The nUMI of myeloid cells; **e** Six cell subtypes of myeloid cells; **f** GSVA analysis of hallmark pathways in macrophages between GGN-ADC and SADC; **g** The expression of key genes in macrophages (GGN-ADC vs SADC); **h** tSNE plotting of 2229 mast cells; **i** The sample origin of mast cells; **j** The marker genes expression in each cluster; **k** GSVA analysis of hallmark pathways in mast cells between GGN-ADC and SADC.

A total of 13,258 myeloid cells were detected in this study. Of them, 8177 myeloid cells were derived from GGN-ADC samples, which accounted for 24.49% on average of GGN-ADC-derived cells in each sample, and 5081 (38.3%) cells were derived from all SADC samples, which accounted for 19.13% on average of SADC-derived cells in each sample, showing no significant difference (*P* = 0.360). The 13,258 myeloid cells were divided into 11 clusters (Fig. 7a). Each cluster contained the cells derived from both GGN-ADC and SADC samples (Fig. 7b and Supplementary Fig. S13a), and almost all clusters contained the cells derived from all ten patients (Fig. 7c and Supplementary Fig. S13b). The mean amount of UMI in myeloid cells derived from GGN-ADC was more than SADC (9183 vs 7680) (Fig. 7d). Reclustering revealed macrophages (clusters 0 and 1, CD163), Langerhans cells (clusters 2, 4, and 5, FCER1A), cross-presenting dendritic cells (DCs) (cluster 9, CLEC9A), granulocytes (cluster 6, S100A12), tumor-associated cells (clusters 0–10, IFITM3), and lung-associated cells (clusters 0–10, RGCC) (Fig. 7e).

Macrophages are one of the important components of the innate immune system. They are involved in phagocytosis, antigen presentation, defense, repair, and metabolism. We therefore focused on the biological functions of various factors, which play important roles in physiological processes such as inflammatory macrophages. We compared the hallmark pathways between the GGN-ADC-derived and SADC-derived macrophages (Fig. 7f). The fat digestion and absorption pathways were mostly significantly increased. The effect of these pathways on macrophage activation need to be further studied. Notably, the NF-kB^33^, PI3K/AKT^34,35^, notch signaling^36^, and mTOR signaling^37^ pathways were also activated, suggesting that macrophages tended to become M1 polarized in GGN-ADCs. In addition, HLA-DR, as the common surface biomarker of M1 macrophage^38^, was overexpressed in macrophages derived from GGN-ADC. Macrophages derived from GGN-ADC also secreted more proinflammatory cytokines, such as IL-1 and TNF-α, which could recruit CTLs to attack cancer cells (all *P* < 0.001). In addition, macrophages derived from GGN-ADCs secreted more chemokines, such as CCL4 (*P* < 0.001; Fig. 7g). Macrophages therefore showed a strong ability to promote inflammation, and played an important role in host immune response to tumor cells in GGN-ADC.

### Mast cells were more enriched in GGN-ADC

With 2229 mast cells detected, 1647 (4.8%) cells originated from GGN-ADC tissues and 582 (2.2%) cells from SADC tissues. The GGN-ADC tissues were more enriched with mast cells when compared to SADC tissues (Fig. 7i and Supplementary Fig. S13c). These mast cells were grouped into three clusters (cluster 0, VWA5A; cluster 1, KLRB1; and cluster 2, CYBB) (Fig. 7h, j). The cells of cluster 0 accounted for more than 90% of all mast cells.

GSVA analyses showed that the chemokine signaling pathway was the most significantly activated in GGN-ADC (Fig. 7k). The JAK-STAT, PI3K/AKT, Notch, and mTOR signaling pathways were also significantly upregulated in mast cells, which played a central role in the inflammatory response associated with cancer^39^. Pathway-related angiogenesis such as the Rap1 signaling, HIF-1 signaling, and VEGF signaling pathways were activated, indicating that mast cells in GGN-ADC promoted tumor angiogenesis. There is increasing evidence to support the view that angiogenesis and inflammation are mutually dependent. During inflammatory reactions, immune cells synthesize and secrete pro-angiogenic factors that promote neovascularization. The newly formed vascular supply contributes to the perpetuation of inflammation by promoting the migration of inflammatory cells to the site of inflammation^40^. This may explain the greater ability of the mast cell-mediated angiogenesis in GGN-ADCs.

### Validation

**Fig. 8:**
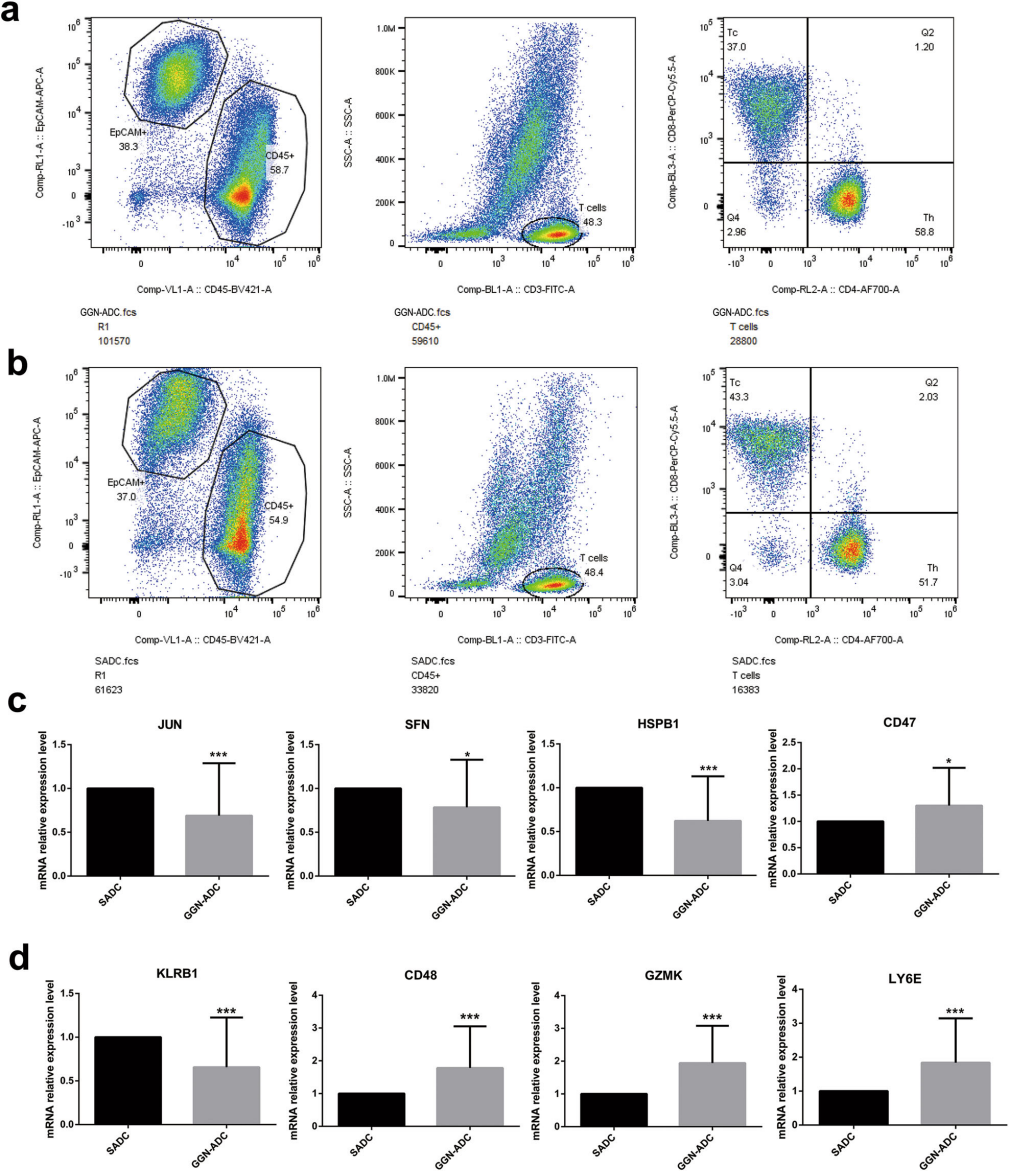
Validation of the key genes expression in cancer cells and CD8+ T cells. **a, b** Cancer cells and CD8+ T cells were identified and sorted by flow cytometry in GGN-ADC and SADC, respectively; **c** The mRNA relative expression level of JUN (*P* < 0.001), SFN (*P* = 0.02), HSPB1 (*P* < 0.001) and CD47 (*P* = 0.014) in cancer cells; **d** The mRNA relative expression level of KLRB1 (*P* < 0.001), CD48 (*P* < 0.001), GZMK (*P* < 0.001) and LY6E (*P* < 0.001) in CD8+ T cells.

The 12 GGN-ADC and 12 SADC samples were used to prepare single-cell suspensions. Flow cytometry was performed to identify and collect the cancer cells marked by EPCAM, T cells marked by CD3D, CD8+ T cells marked by CD8A, and CD4+ T cells marked by CD4 in the GGN-ADC and SADC samples (Fig. 8a, b). The quantitative real-time polymerase chain reaction (qRT-PCR) was performed to validate the difference of gene expression levels in cancer cells and CD8+ T cells (Fig. 8c). The expression levels of JUN, SFN, HSPB1, and CD47 in cancer cells and the expression levels of KLRB1, CD48, GZMK, and LY6E in CD8+ T cells were consistent with the scRNA-seq results (Fig. 8c, d).

## Discussion

GGN is an inert pathological subtype that progresses slowly, and the guidelines for management of GGNs differ from solid nodules. With the increased use of CT, the percentages of GGNs detected in lung cancer patients is increasing. There are currently no definite surgical indications for GGNs. The more accepted surgical indications for GGNs are changes in the property of GGNs, such as an increase in size of lesions, pleural depression, or changes in GGN components (pure GGN becomes mixed GGN or mixed GGN becomes solid nodules). Recent studies reported that patients with GGN had excellent long-term overall survival, which suggested that these patients had low-grade malignancies and excellent prognoses^5,41^. The majority of patients with GGNs receive routine CT examinations. The main management for patients with GGNs is routine CT surveillance because the majority of GGNs do not progress to malignancy, and even if they did, a “delay” in surgery does not usually result in poorer survival^42–44^. However, we observed that GGNs were characterized by slow growth, although the mechanism is unknown. Our research aim was to characterize the molecular mechanism of slow growth of GGNs, and to obtain a more comprehensive understanding of this pathological subtype. GGNs are largely between a benign and malignant lesion. Exploring the characteristics of GGN cancer cells and stromal cells contributes to our understanding of the mechanism of the occurrence and development of lung cancer.

Tumor tissue is mainly composed of tumor cells and a large amount of stromal cells that constitute the TME. The existence of stromal cells would greatly influence the sequence analysis of cancer cells if analyzed by traditional gene chip and second-generation sequencing. However, scRNA-seq can overcome the inherent problems of these methodologies^8^. By using unbiased scRNA-seq techniques, we presented a comprehensive profile of cells derived from GGN-ADC and SADC tissues at the single-cell level. Using subtype-specific marker genes, we identified eight cell types from GGN-ADC and SADC samples, including cancer cells and seven types of stromal cells. By analyzing the differences of enriched signaling pathways, the important genes and transcriptional factors of cancer cells and each stromal cell type between the GGN-ADC and SADC groups, we confirmed many important observations on cancer cells and the TME.

Tumor cell activity and differentiation degree are associated with the ability of tumor proliferation, invasion, and metastasis. In our study, cancer cells derived from GGN-ADC and SADC exhibited different biological properties. Overall, cancer cells derived from GGN-ADC had a low-grade malignancy compared with those from SADC. We identified 37 stromal subtypes, including different types of fibroblasts, endothelial cells, and tumor-infiltrating immune cells. Each cell type showed divergent pathway activities between GGN-ADC and SADC, suggesting that they represented distinct biological entities.

The TME refers to the local steady state environment associated with tumorigenesis and metastasis. The interaction between tumor cells and the TME determines tumor progression. In the process of tumor development, the immune system plays two different roles. These immune cells have a natural anti-tumor effect at the beginning of tumor invasion, but also have a tumor-promoting phenotype during tumor progression, assisting tumor immune escape and metastasis. We thought that the TME exerted tumor suppression effects in our study because we selected the tumor at the early stages of growth. In addition to various types of inflammatory/immune cells, the TME also contains stromal cells such as fibroblasts and endothelial cells. These cells not only provide protection and support for the initiation and development of tumors, but also actively participate in tumor progression. Our results showed that endothelial cells and fibroblasts in GGNs exerted weaker effects on promoting tumor progress than those in SADC.

Our study adopted 10× Genomics high-throughput scRNA-seq analyses to compare the properties of GGN-ADC and SADC. High-throughput scRNA-seq has substantially increased the number of detected cells per sample compared with traditional scRNA-seq (from dozens to thousands). Compared with second-generation sequencing techniques, high-throughput scRNA-seq single cell sequencing can detect changes at the molecular level in each cell more effectively. In addition, for the first time, we used flow cytometry and qRT-PCR to analyze the expression level changes of key molecules in the cell subtypes, to validate the results of high-throughput scRNA-seq analyses. The scRNA-seq techniques have previously been applied for lung cancer^8^, chronic myeloid leukemia^45^, and hepatocellular carcinoma studies^46^, and we believe that high-throughput scRNA-seq techniques will be more widely used in the future.

There were some limitations in this study. First, because GGN-ADC and SADC are the same pathological type, tSNE plotting failed to reveal separation between the two groups. We therefore only analyzed the differences in biological functions and key molecular expression levels of each cell type/subtype by groups. Second, all cell types, subtypes, and phenotypes could not possibly be fully described in a single report, so only some key observations emerged in our studies, which will be further investigated on a more comprehensive and deeper level in the future. Third, when obtaining the specimens, it is difficult to distinguish the GGO part and a solid part in the same tumor. In the further study, we intend to obtain GGO tissues and solid tissues from the same large mixed GGN-ADC to investigate the differences between the GGO part and a solid part in the same tumor.

Using the scRNA-seq technique, we analyzed the possible causes of slow growth of GGN-ADC at the genetic and molecular levels, and concluded that cancer cells and the TME together contributed to the growth of GGN-ADC when compared with SADC. Understanding the biological properties and function of cancer cells and the TME is critical to effectively inhibiting cancer progression.

## Materials and methods

### Patients

This study was approved by the Ethics Committee of Zhongshan Hospital, Fudan University, China (B2018–137R), and complied with all relevant ethical regulations. Written informed consent was obtained from all patients.

Five patients with GGN-ADCs and an equal number of patients with SADCs were included in the current study for scRNA-seq analyses. In addition, 12 GGN-ADC samples and 12 SADC samples were used for flow cytometry and qRT-PCR analyses. Patient information and clinical characteristics were also collected.

### Preparation of single-cell suspensions

For each patient, a small sample of the tumor was obtained after resection in the operating room and used to prepare a single-cell suspension. Tumor tissues can be dissociated into single-cell suspensions by combining mechanical dissociation with enzymatic degradation of the extracellular matrix, which maintains the structural integrity of tissues. The tumor tissue was enzymatically digested using a Tumor Dissociation Kit (Miltenyi Biotec, Gladbach, Germany) according to the manufacturer’s instructions. Briefly, the tissue sample was cut into small pieces, and the pieces were transferred to a gentle MACS C Tube containing 4.7 mL Dulbecco’s Modified Essential Medium, 200 µL enzyme H, 100 µL enzyme R, and 25 µL enzyme A. The gentle MACS™ Dissociator was used three times for the mechanical dissociation steps. The sample was then incubated for 30 min at 37 °C at each dissociation step interval. After dissociation, the sample was passed through a filter with a pore size of 40 μm to remove any remaining large particles from the single cell suspension. The suspension was centrifuged at 300 × *g* for 7 min and the supernatant was completely removed. Next, Red Blood Cell Lysis Solution (10×) (Sigma-Aldrich, St. Louis, MO, USA) was used to remove erythrocytes. Briefly, 1× lysis solution was added to the centrifuge tube that contained the remaining cell pellet. The cell suspension was then incubated in the dark for 15 min. To remove dead cells, a Dead Cell Removal Kit (Miltenyi Biotec) was used to ensure a cell viability >90%.

### ScRNA-seq

ScRNA-seq libraries were prepared using a Chromium Single cell 3′ Reagent kit, version 2, according to the manufacturer’s protocol. Single-cell suspensions were loaded on the Chromium Single Cell Controller Instrument (10× Genomics, Pleasanton, CA, USA) to generate single cell gel beads in emulsions (GEMs). Briefly, 1 × 106 single cells were suspended in calcium- and magnesium-free phosphate-buffered saline (PBS) containing 0.04% w/v bovine serum albumin. About 10,000 cells were added to each channel with a targeted cell recovery estimate of 8000 cells. After generation of GEMs, reverse transcription reactions used barcoded full-length cDNA followed by the disruption of emulsions using the recovery agent and cDNA clean up with DynaBeads Myone Silane Beads (Thermo Fisher Scientific, Waltham, MA, USA). The cDNA was then amplified by PCR with appropriate cycles, which depended on the recovery of cells. Subsequently, the amplified cDNA was fragmented, end-repaired, A-tailed, index adapter ligated, and library amplification. Then these libraries were sequenced on the Illumina sequencing platform (HiSeq X Ten; Illumina, San Diego, CA, USA) and 150 bp paired-end reads were generated.

### ScRNA-seq data preprocessing

The Cell Ranger software pipeline (version 3.0.0) provided by 10× Genomics was used to demultiplex cellular barcodes, map reads to the genome, and align transcriptomes using the STAR aligner, and down-sample reads as required to generate normalized aggregate data across samples, producing a matrix of gene counts versus cells. We processed the unique molecular identifier (UMI) count matrix using the R package Seurat (version 2.3.4).

To remove low quality cells and likely multiplet captures, which is a major concern in microdroplet-based experiments, we applied a criteria to filter out cells with UMI/gene numbers out of the limit of mean values ± two-fold of SD, assuming a Gaussian distribution of each cell UMI/gene numbers. Following visual inspection of the distribution of cells by the fraction of mitochondrial genes expressed, we further discarded low quality cells where >10% of the counts belonged to mitochondrial genes. After applying these quality control criteria, 60,459 single cells and 33,694 genes in total remained, and were included in downstream analyses. Library size normalization was performed in Seurat on the filtered matrix to obtain the normalized counts.

Initial CNVs for each region were estimated by inferCNV R package^47^. The CNV of total cell types were calculated by expression level from scRNA-seq data for each cell. The CNV score of each cell was calculated as quadratic sum of CNV region.

Top variable genes across single cells were identified using the method described by Macosko et al.^48^. Briefly, the average expression and dispersion were calculated for each gene, and the genes were subsequently placed into 13 bins based on expression. Principal component analysis was performed to reduce the dimensionality on the log transformed gene-barcode matrices of top variable genes. Cells were clustered based on a graph-based clustering approach, and were visualized in two dimensions using tSNE. Likelihood ratio tests that simultaneously tested for changes in mean expression and in the percentage of expressed cells was used to identify significantly differentially expressed genes between clusters. We used the SingleR R package, a novel computational method for unbiased cell type recognition of scRNA-seq, with two reference transcriptomic datasets of “Human Primary Cell Atlas”^49^ and “Human Cell Landscape” (http://bis.zju.edu.cn/HCL/index.htmL) to independently infer the cell of origin of each of the single cells and to identify cell types. In order to show the difference of the overall expression of the genes in the sample, we used the gene expression values normalized by Seurat for analysis, and screened the genes in the signaling pathway with average expression greater than 0.1 in at least one sample. *Z*-score was used to show the pathway strength and gene expression. GSVA analyses showed the significantly different signaling pathways. GSVA converted the expression matrix into a path enrichment score matrix, and then used the lmFit analysis of the limma package to obtain the different pathways. *P* value was calculated by the limma package. FDR was also provided. Library size normalization was performed using the NormalizeData function in the Seurat R package. Specifically, the global-scaling normalization method “LogNormalize” normalized the gene expression measurements for each cell by the total expression, multiplied by a scaling factor (10,000 by default), and the results were logtransformed.

To assess which transcription factors were responsible for the differences in expression between GGN-ADC and SADC cancer cells, we applied SCENIC analysis. The SCENIC analysis was run using the motifs database for RcisTarget and GRNboost (SCENIC version 1.1.2.2, which corresponds to RcisTarget 1.2.1 and AUCell 1.4.1) with the default parameters^50^.

### Validation

Single cells were suspended in PBS with 3% fetal bovine serum. Nonspecific antibody binding was blocked by incubation with 20 μg/mL human IgG (Sigma-Aldrich) for 15 min, and cells were then incubated with allophycocyanin-conjugated mouse anti-human EPCAM (BD Biosciences, San Jose, CA, USA), BV421-conjugated mouse anti-human CD45 (eBioscience, San Diego, CA, USA), FITC-conjugated mouse anti-human CD3 (eBioscience), PerCP-Cy5.5-conjugated mouse anti-human CD8A (eBioscience), and AF700-conjugated mouse anti-human CD4 (eBioscience) for 30 min on ice. Stained cells were quantitated using a Fortessa analyzer (BD Biosciences) and isolated with a FACSAria II (BD Biosciences). FlowJo software (TreeStar, Woodburn, OR, USA) was used to generate the flow described above. Sorted cells were immediately subjected to RNA extraction and reverse transcription using a kit (Takara, Kusatsu, Japan) prior to qRT-PCR analyses. The primers were as follows: JUN-forward: 5′-TCCAAGTGCCGAAAAAGGAAG-3′; JUN-reverse: 5′-CGAGTTCTGAGCTTTCAAGGT-3′; SFN-forward: 5′-TGACGACAAGAAGCGCATCAT-3′; SFN-reverse: 5′-GTAGTGGAAGACGGAAAAGTTCA-3′; HSPB1-forward: 5′-TGGACCCCACCCAAGTTTC-3′; HSPB1-reverse: 5′-CGGCAGTCTCATCGGATTTT-3′; CD47-forward: 5′-TCCGGTGGTATGGATGAGAAA-3′; CD47-reverse: 5′-ACCAAGGCCAGTAGCATTCTT-3′; GZMB-forward: 5′-CCCTGGGAAAACACTCACACA-3′; GZMB-reverse: 5′-GCACAACTCAATGGTACTGTCG-3′. The diagrams were generated by Graphpad software (GraphPad Prism 6.01, Inc., La Jolla, United States).

## Supplementary information

Supplementary information

## Supplementary information

**Supplementary Information** accompanies the paper at (10.1038/s41421-020-00200-x).
