# Supplementary material for: Single-cell transcriptome atlas of lung adenocarcinoma featured with ground glass nodules
Source: Cell Discov. 2020 Oct 6;6:69. doi: 10.1038/s41421-020-00200-x (PMC7536439; doi:10.1038/s41421-020-00200-x)
Supplement: Supplementary file 1 — Supplementary information [file 41421_2020_200_MOESM1_ESM.pdf]

Supplementary Figures

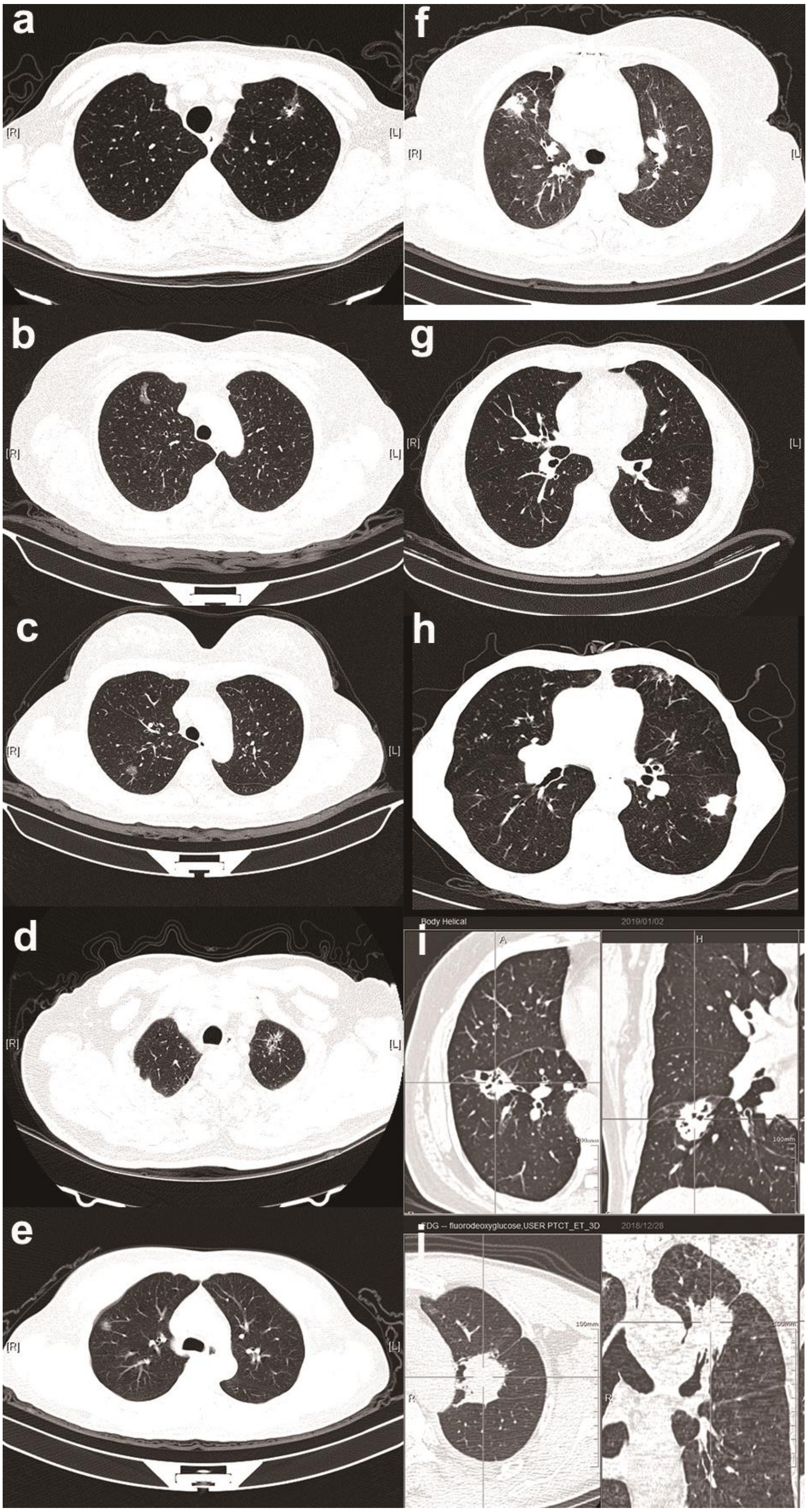

Fig. S1. The imaging performance of the five GGN-ADC patients (a-e) and SADC patients (f-j).

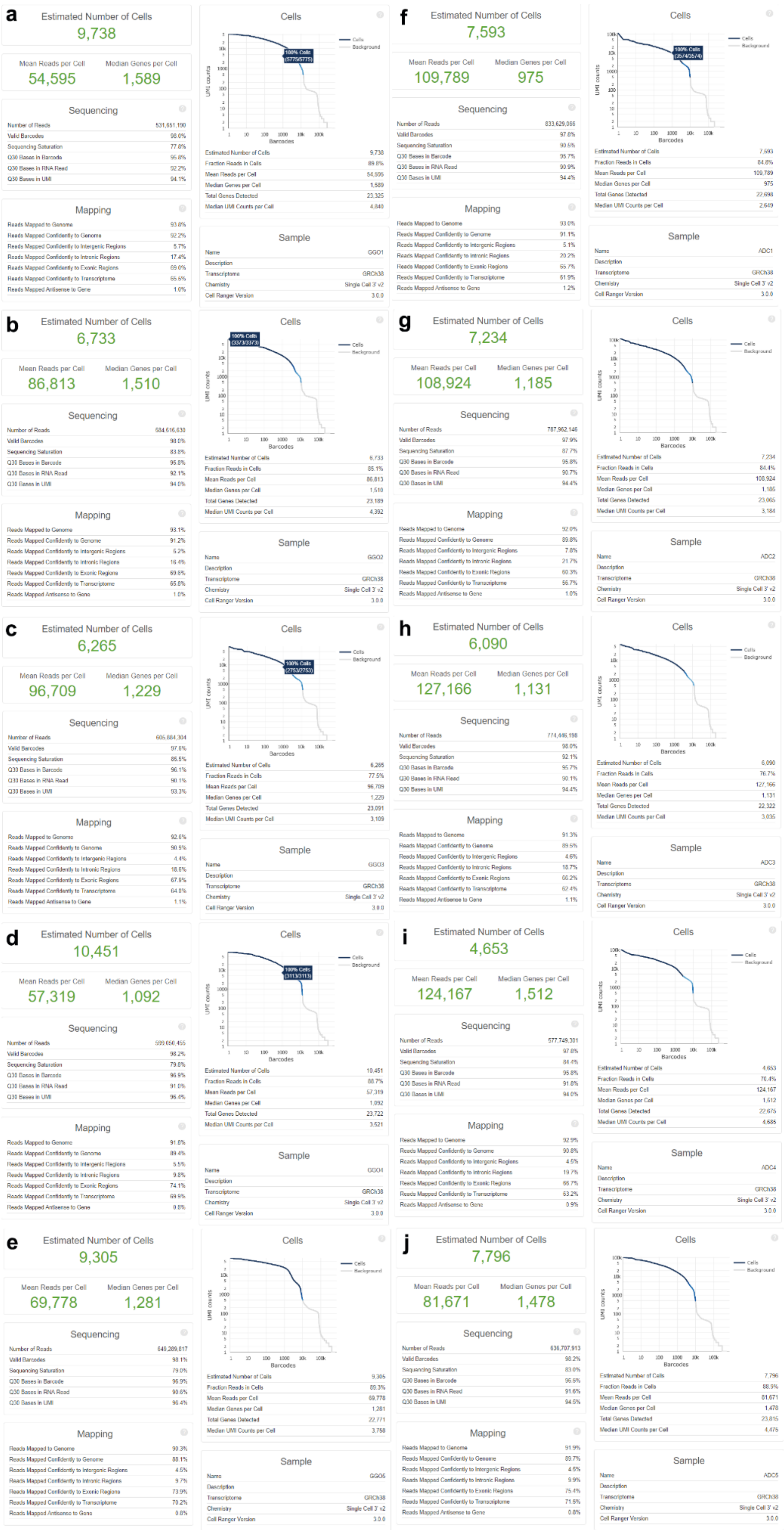

Fig. S2. Quality control metrics of the five GGN-ADC samples (a-e) and SADC samples (f-j).

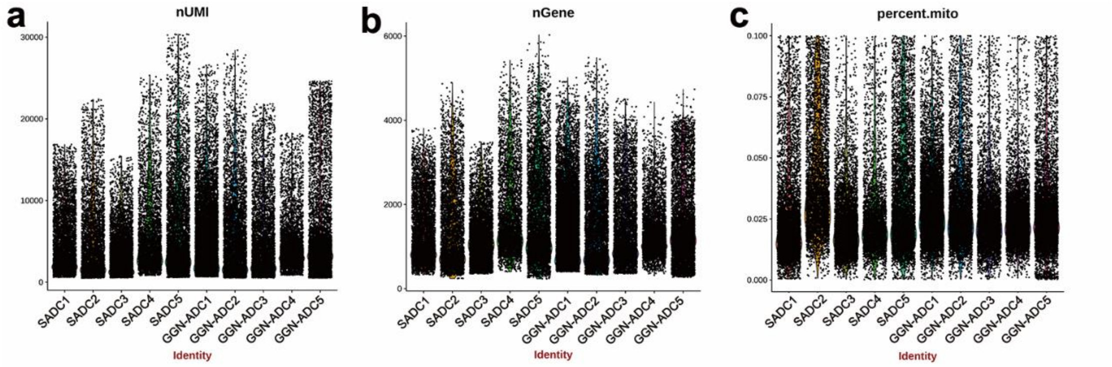

Fig. S3. The distribution of the nUMI (a), nGene (b), and the percentage of mitochondrial counts (c) of the samples.

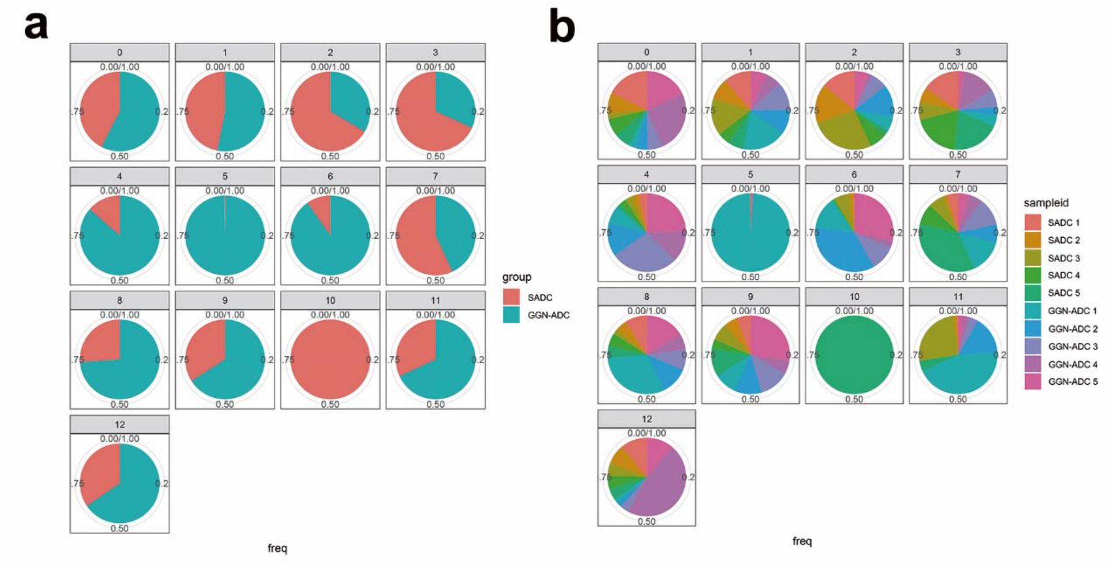

Fig. S4. The cluster origin of all detected cells. (a) Group origin; (b) Sample origin.

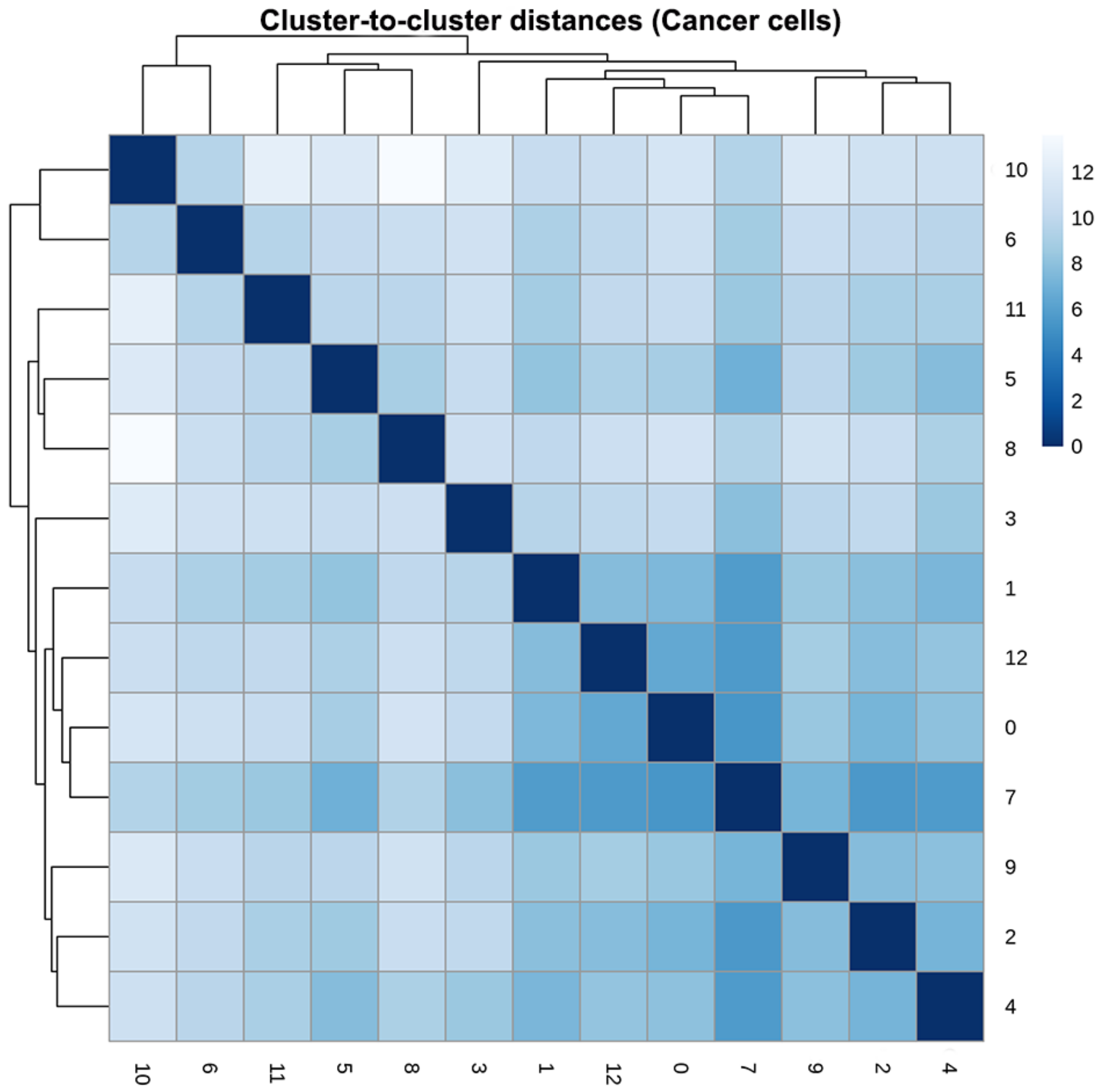

Fig. S5. The cluster-to-cluster distance of cancer cells.

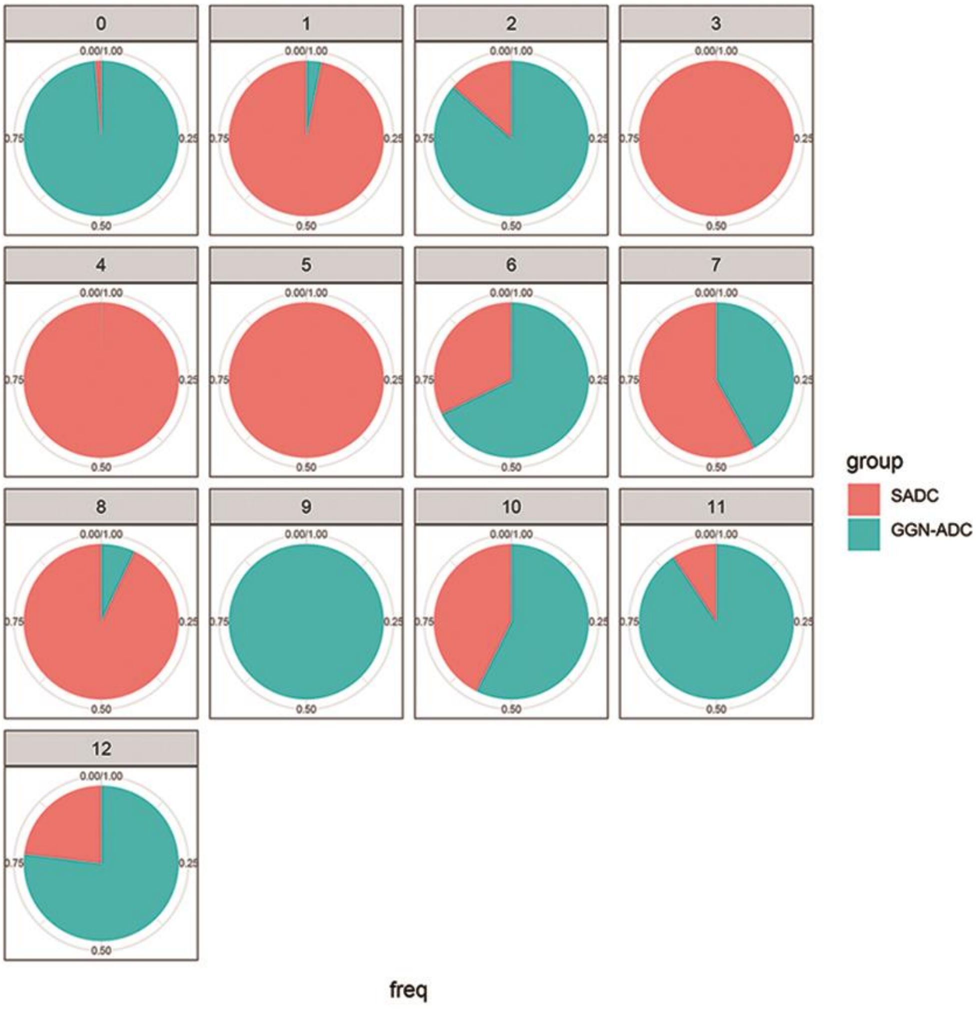

Fig. S6. The group-by-group tSNE showed that the clusters from SADC were highly patient-specific.

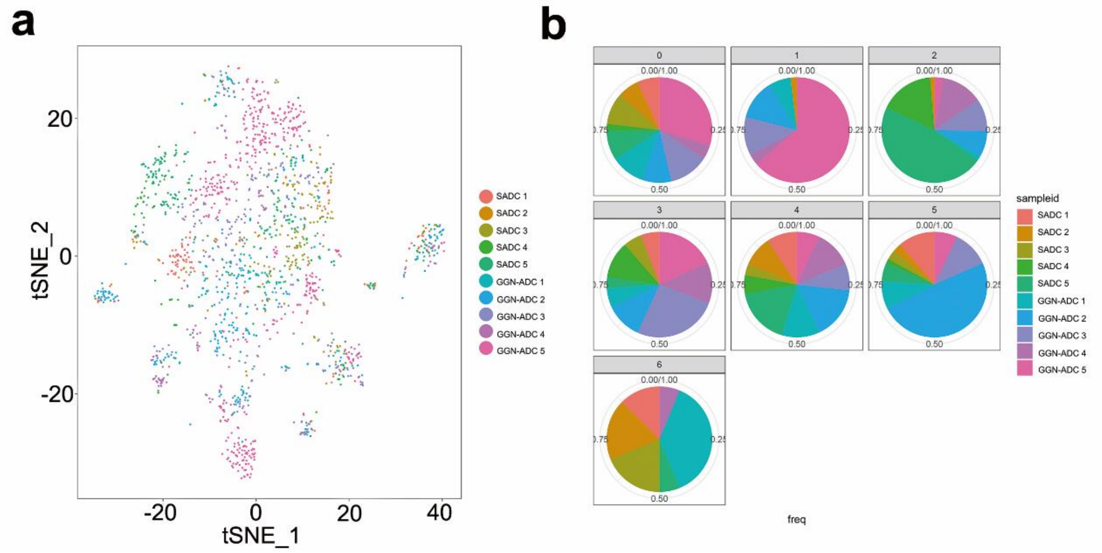

Fig. S7. The patient origin of the endothelial cells.

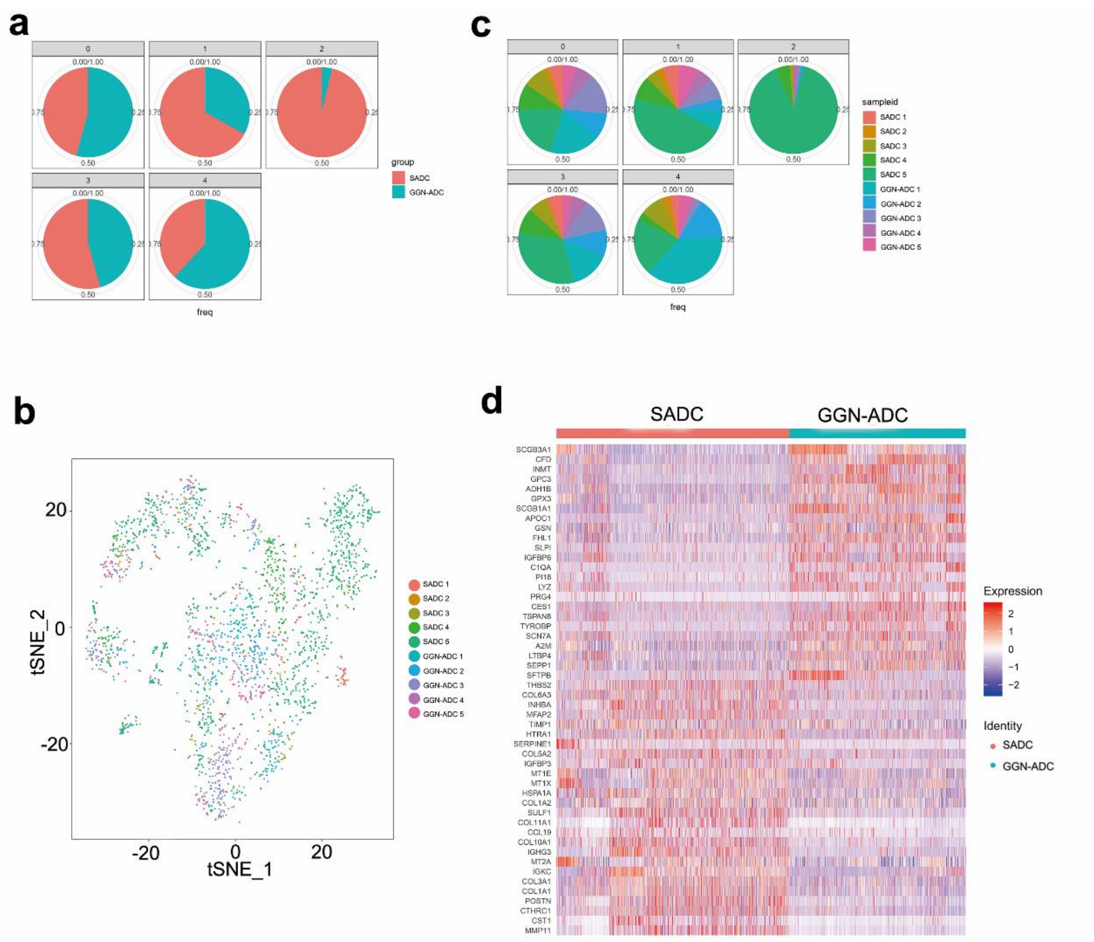

Fig. S8. Fibroblasts. The group origin of the clusters (a); The sample origin of the clusters (b, c); The differential expressed genes (d).

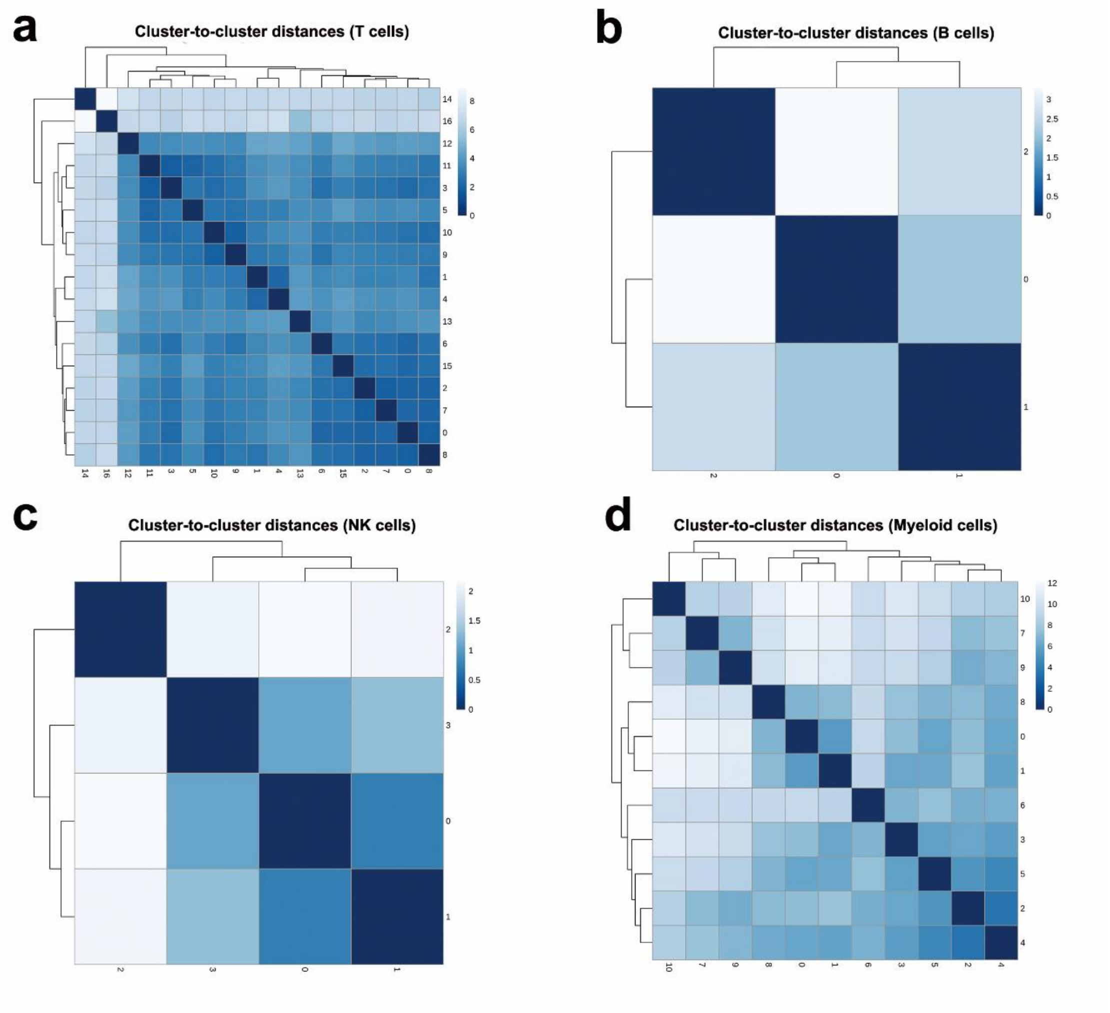

Fig. S9. The cluster-to-cluster distances of the T cells (a), B cells (b), NK cells (c), myeloid cells (d).

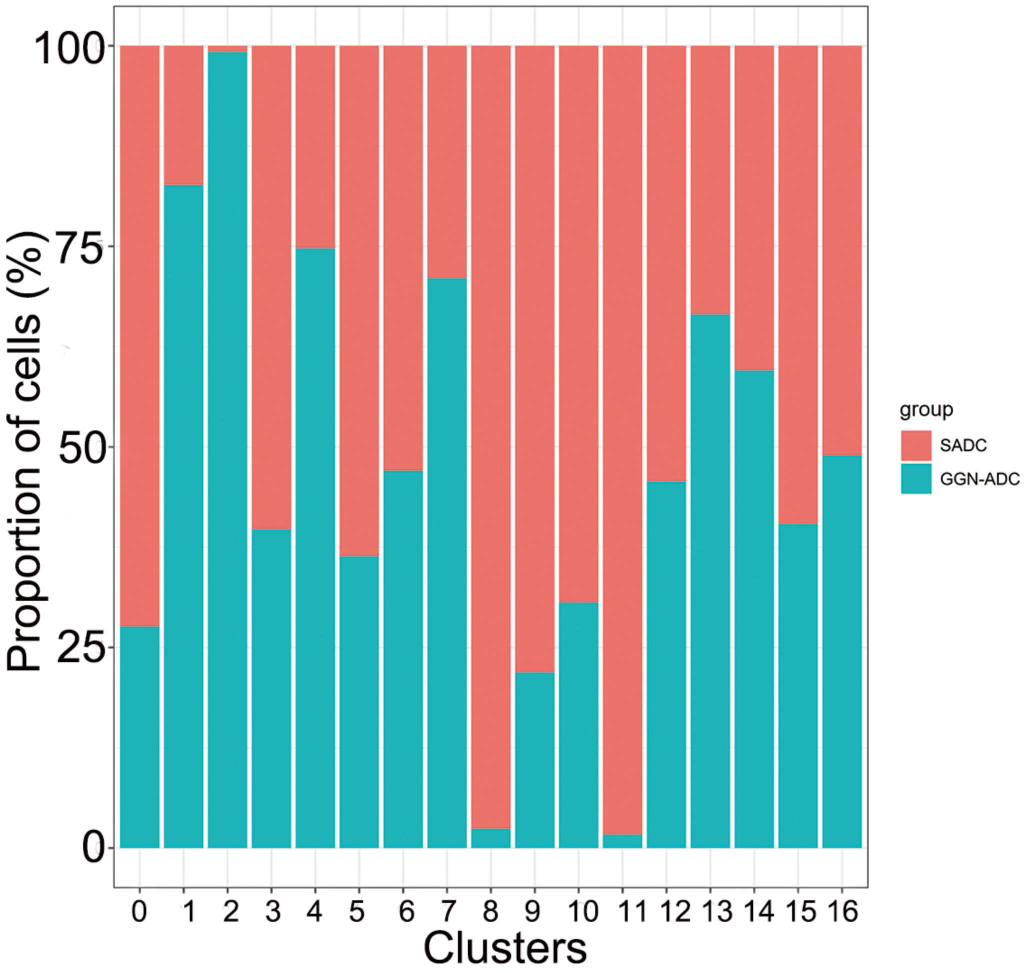

Fig. S10. The origin of the clusters in T cells.

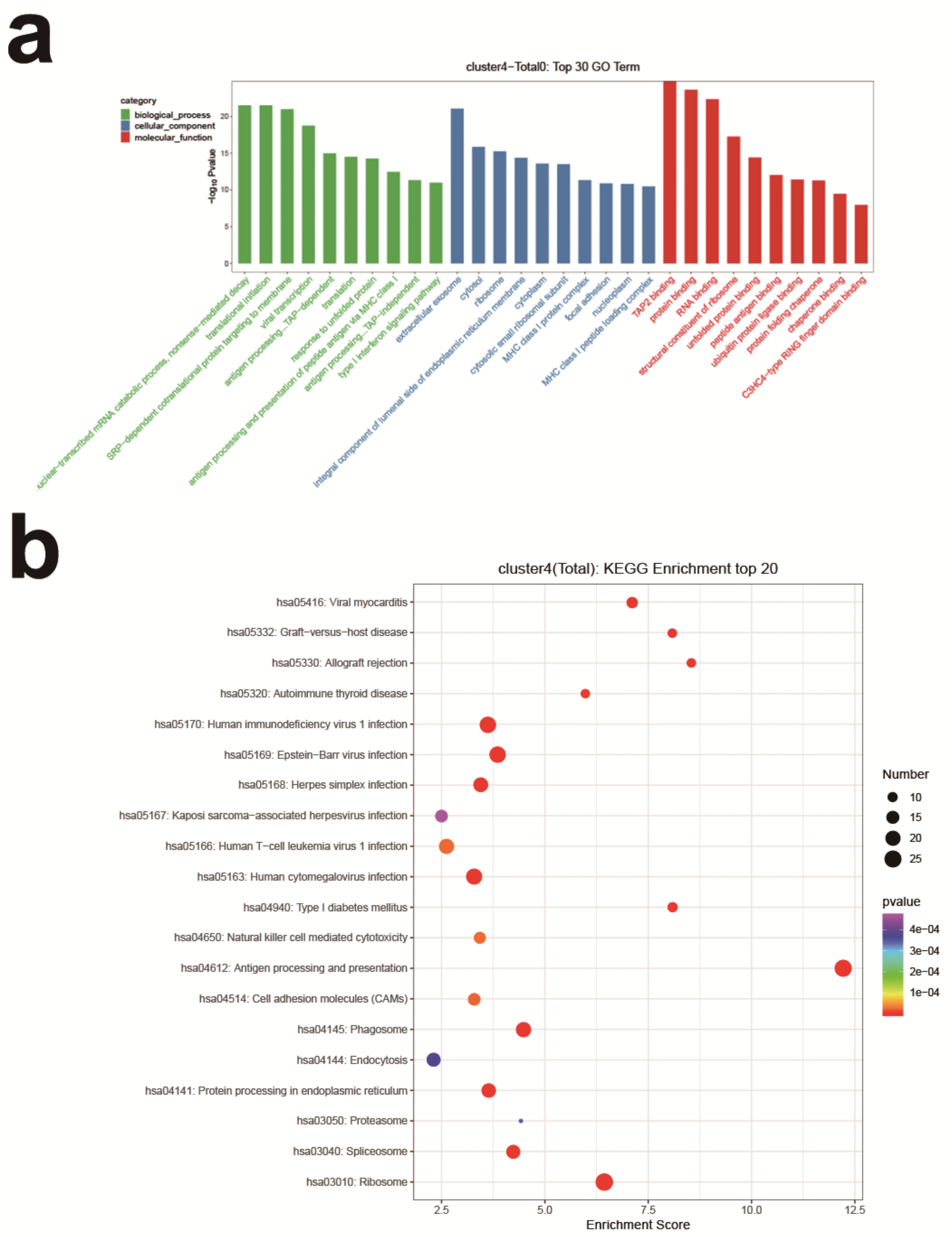

Fig. S11. Enrichment of functions and signaling pathways of the differential expressed genes. (a) GO analysis; (b) KEGG analysis.

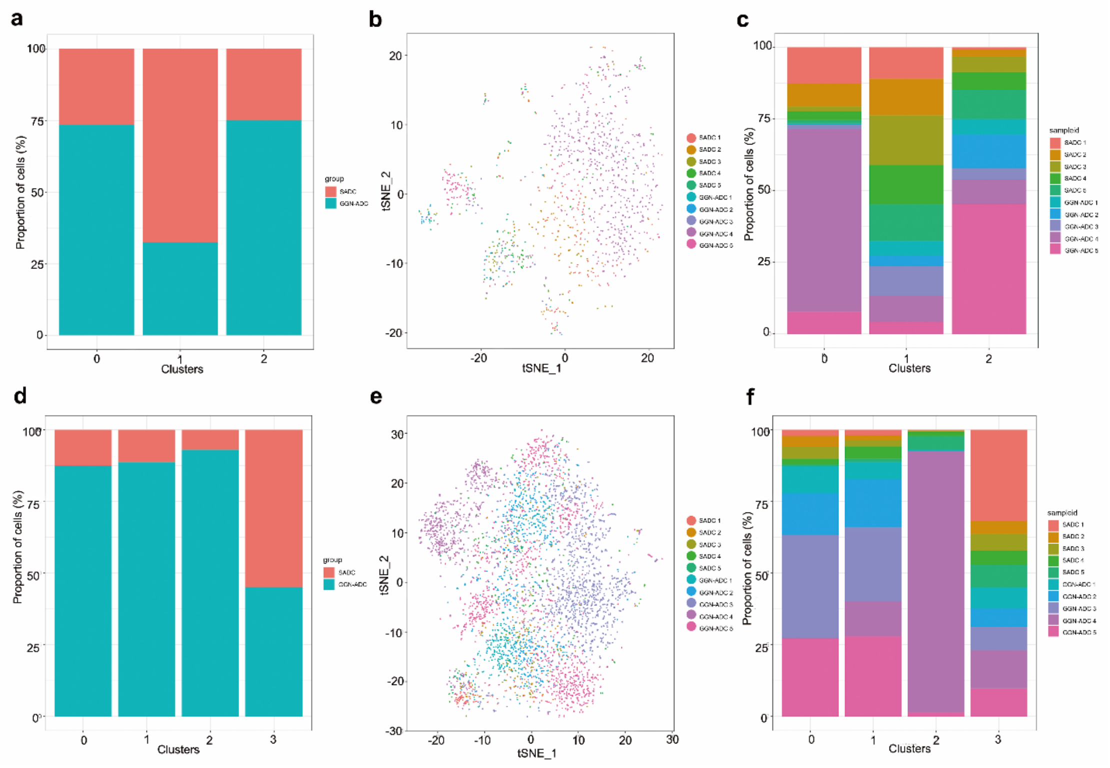

Fig. S12. The group origin of the B cells clusters (a); The patient origin of the B cells clusters (b, c); The group origin of the NK cells clusters (d); The patient origin of the NK cells clusters (e, f).

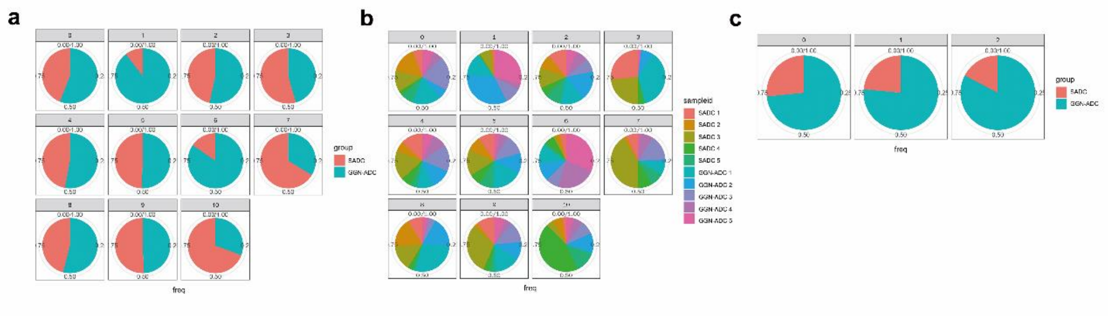

Fig. S13. The group origin of the macrophage clusters (a); The patient origin of the macrophage clusters (b); The group origin of the mast cell clusters (c).
